# Environmental and ethnic differences in short-term risk of first acute myocardial infarction: a prospective multiethnic cohort protocol

**DOI:** 10.3389/fpubh.2026.1848166

**Published:** 2026-08-12

**Authors:** Bowen Wang, Zhan Zhang, Weijie Lu, Gang Wang, Ke Chen, Wending Gu, Yanbiao Shu, Juan Wang, Tiancheng Zhang, Yifan Zhang, Rong Wang, Lei Xu, Ping Xie

**Affiliations:** 1 Department of Cardiology, Gansu Provincial Hospital, Lanzhou, China; 2 Gansu University of Chinese Medicine, Lanzhou, China; 3 Lanzhou University, Lanzhou, China; 4 Department of Nuclear Medicine, Gansu Provincial Hospital, Lanzhou, China; 5 Vanke School of Public Health, Tsinghua University, Beijing, China; 6 Institute for Healthy China, Tsinghua University, Beijing, China

**Keywords:** environmental, ethnic, first acute myocardial infarction, protocol, short-term risk

## Abstract

**Background:**

Acute myocardial infarction (AMI) remains a leading cause of mortality worldwide, with substantial variation across populations shaped by environmental conditions, geographic context, and healthcare accessibility. However, current risk assessment approaches based on baseline measurements may not adequately capture short-term risk or population heterogeneity.

**Methods and analysis:**

This multicentre prospective cohort study will recruit 2,340 adults aged 20–85 years with an estimated 10-year atherosclerotic cardiovascular disease risk ≥20%. Participants will be enrolled from five hospitals in Gansu Province, China, representing variation in ethnicity, altitude, and healthcare access, including Han, Hui, Tibetan, and Kazakh populations. Baseline assessments will include standardized questionnaires, clinical evaluation, and cardiometabolic profiling. Participants will be followed every 3 months for 24 months, with repeated measurements of behavioral, physiological, and biomarker indicators, including wearable electrocardiographic monitoring. Environmental exposures and healthcare accessibility will be assigned using geospatial data. The primary outcome is first incident AMI. Time-to-event models with time-varying variables will be used to examine how within-individual changes in risk indicators are associated with AMI across environmental and ethnic contexts.

**Ethics and dissemination:**

The study has been approved by the Ethics Committee of Gansu Provincial Hospital. Written informed consent will be obtained from all participants. Findings will be disseminated through peer-reviewed publications and scientific presentations.

**Clinical trial registration:**

ChiCTR2500104518.

## Introduction

Acute myocardial infarction (AMI) remains a leading cause of mortality worldwide and reflects a major burden of cardiovascular disease at the population level (1). Beyond individual risk factors, environmental conditions and healthcare accessibility play an important role in shaping cardiovascular outcomes. Regional variation in air pollution, climate, altitude, and healthcare resources contributes to differences in cardiovascular risk, highlighting the importance of contextual determinants in public health (2–4).

Ethnic differences further contribute to heterogeneity in cardiovascular risk. Variations in lifestyle, dietary patterns, and social conditions across ethnic groups have been associated with differences in cardiovascular risk profiles and disease burden (5, 6). In western China, populations including Han, Hui, Tibetan, and Kazakh communities are exposed to distinct environmental and social contexts, resulting in heterogeneous cardiometabolic risk patterns (7–9). High-altitude exposure in Tibetan populations is associated with specific physiological adaptations that may influence cardiovascular risk (10).

Despite these insights, most existing studies have focused on long-term risk estimation or cross-sectional comparisons. Commonly used tools, such as the Framingham Risk Score and the China-PAR model, are based on baseline measurements and assume relatively stable risk over time (11–13). Although recent studies have incorporated longitudinal data and time-varying models (14, 15), environmental exposure, healthcare accessibility, and ethnic context are rarely examined simultaneously. As a result, it remains unclear whether short-term risk patterns preceding first AMI differ across populations.

To address this gap, a prospective study integrating environmental exposure, ethnic background, healthcare accessibility, and repeated multidimensional assessment is needed. The present study aims to investigate the short-term risk of first AMI in a multiethnic cohort and to examine how environmental and ethnic differences are associated with variation in short-term cardiovascular risk. By focusing on population-level heterogeneity and within-individual changes over time, this study seeks to provide a more context-sensitive understanding of cardiovascular risk and to inform targeted prevention strategies.

## Method

### Study design

**Figure 1:**
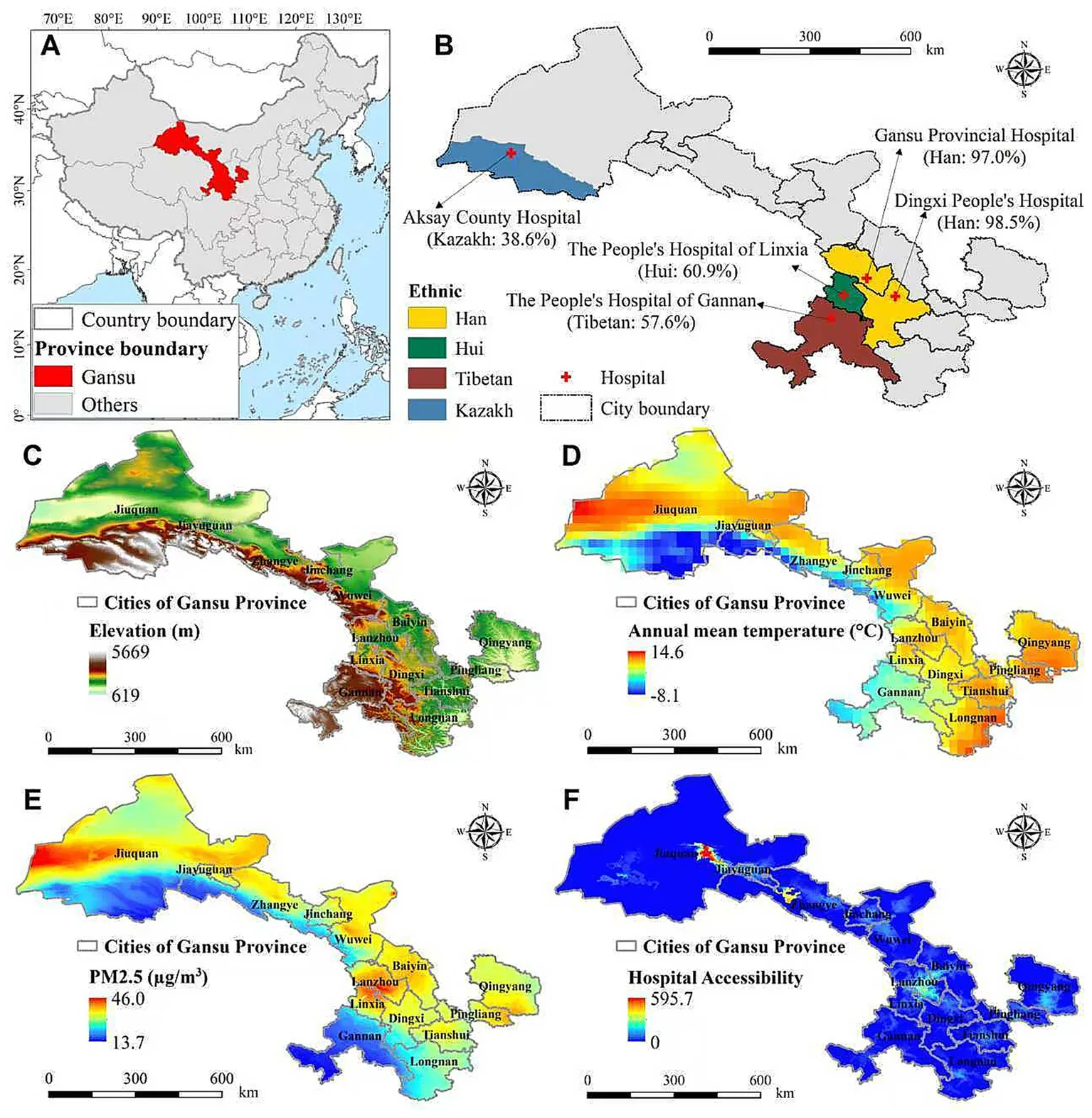
Overview of the study area and environmental determinants. **(A)** Inset map indicating the geographic position of Gansu Province within China. **(B)** Map of Gansu Province showing the locations of the five participating hospitals and the predominant ethnic groups (Han, Hui, Tibetan, Kazakh, Others) with their respective percentages at each site. **(C)** Spatial distribution of elevation (m) across Gansu. **(D)** Spatial distribution of annual mean temperature (°C) across Gansu. **(E)** Spatial distribution of PM2.5 concentration (μg/m³) across Gansu. **(F)** Spatial distribution of hospital accessibility index.

This is a prospective, multicentre, hospital-based cohort study with an embedded nested case–control component conducted across five hospitals in Gansu Province, China (Figure 1). Gansu Province, located in northwestern China (16), provides substantial variation in altitude, ethnicity, environmental exposure, and healthcare accessibility. The study aims to examine variation in short-term risk of AMI across populations exposed to different environmental, geographic, and healthcare conditions in western China. Participating centers include Gansu Provincial Hospital (103°51′E, 36°03′N,predominantly Han population; mean altitude 2,098 m), Aksai Kazakh Autonomous County Hospital (94°20’E, 39°24’N, predominantly Kazakh; 3,289 m), Dingxi People’s Hospital (104°37’E, 35°34’N, predominantly Han; 2,282 m), Linxia People’s Hospital (103°14’E, 35°36’N, predominantly Hui, 2,255 m), and Gannan People’s Hospital (102°91’E, 34°98’N, predominantly Tibetan, 3,369 m). These centers represent distinct combinations of altitude, ethnicity, and healthcare accessibility. Standardized protocols are applied across all sites for recruitment, baseline assessment, follow-up, and outcome adjudication, with centralized coordination and regular cross-site training to ensure consistency.

### Study population

A total of approximately 2,340 adults will be enrolled from five participating centers, representing Han, Hui, Tibetan, and Kazakh populations across regions with varying altitude, environmental exposure, and healthcare accessibility. Recruitment targets are prespecified according to catchment characteristics, with planned enrolment of 900–1,050 Han, 650–750 Hui, 400–500 Tibetan, and 250–350 Kazakh participants. Sample size estimation is based on an event-driven approach (Supplementary Material 1). Assuming a 4% cumulative incidence of first AMI over 24 months, approximately 80 events are expected, supporting multivariable modeling and subgroup analyses.

Participants will be eligible if they: (1) are aged 30–85 years; (2) have an estimated 10-year atherosclerotic cardiovascular disease (ASCVD) risk ≥20% (6); (3) are permanent residents of the study area (≥6 months per year); (4) provide written informed consent; and (5) agree to repeated follow-up and biospecimen collection. Exclusion criteria include: (1) major comorbid conditions such as end-stage renal disease, hepatic failure, or active malignancy; (2) acute illness at enrolment; (3) severe psychiatric disorders; (4) substance abuse; and (5) pregnancy or breastfeeding (17). Loss to follow-up is defined as absence from more than two scheduled visits or permanent discontinuation. Follow-up will be maintained through clinic visits supplemented by telephone or electronic contact where necessary.

### Variables and data collection

#### Baseline variables

**Figure 2:**
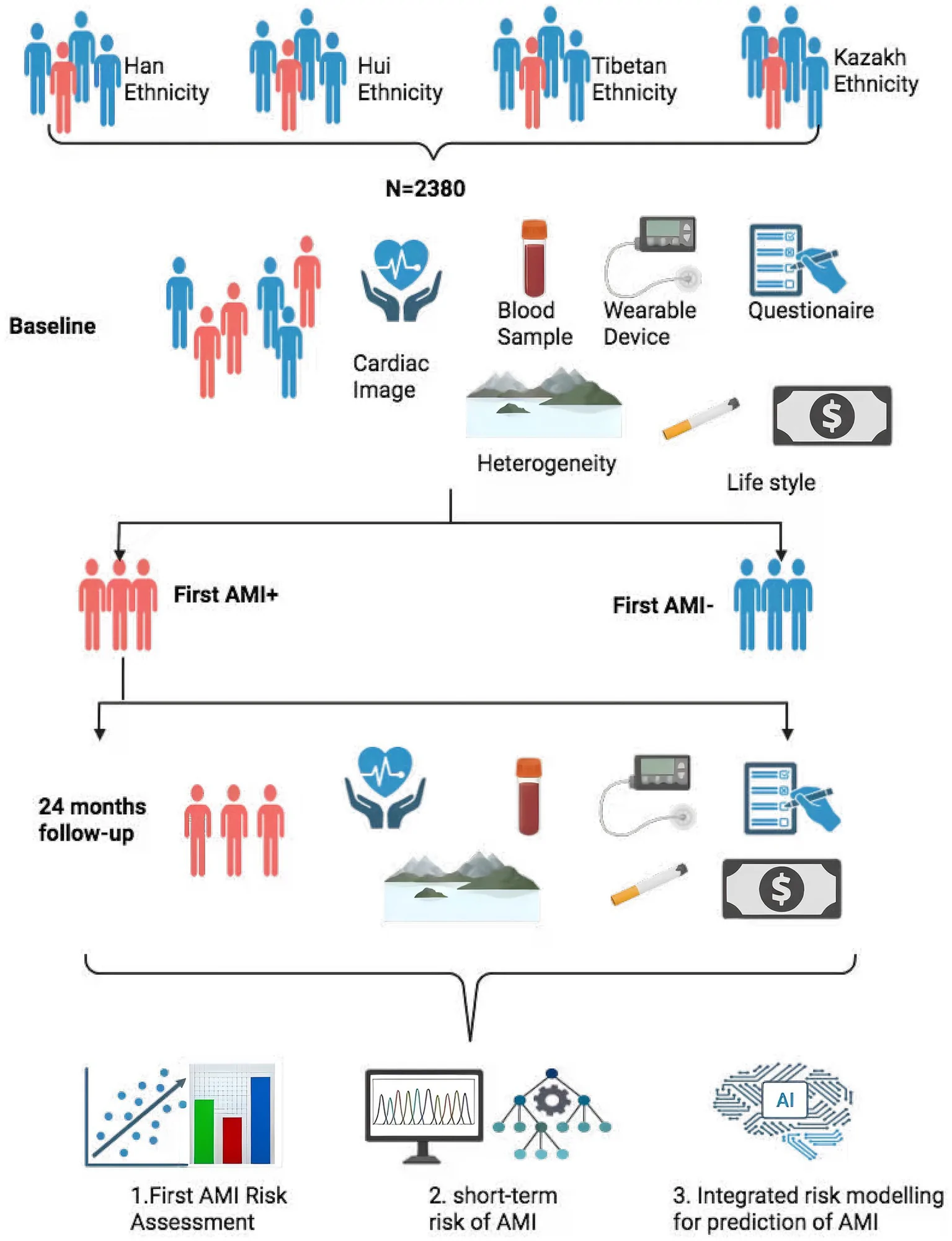
Schematic overview of the study design.

At enrolment, participants undergo standardized assessments including questionnaires, physical examination, laboratory testing, cardiovascular imaging, and physiological monitoring (Table 1; Figure 2). These measurements are designed to capture both individual-level risk factors and population-level variation related to environmental and ethnic contexts. Sociodemographic variables include age, sex, ethnicity, residence type (urban or rural), education level, occupation, and household income. Clinical history captures physician-diagnosed hypertension, diabetes mellitus, dyslipidaemia, prior stroke or transient ischemic attack (TIA), and family history of coronary artery disease (CAD), together with current medication use. Lifestyle-related variables include smoking status, classified as never, former, or current smoking, alcohol consumption, dietary patterns, physical activity level, sleep quality, medication adherence, and psychological status, which may vary across environmental and sociocultural settings. Physiological measurements include systolic blood pressure, diastolic blood pressure, resting heart rate, body mass index, waist circumference, and body fat percentage (18). Laboratory biomarkers include inflammatory and cardiac stress markers, cardiac enzymes, and metabolic indicators reflecting glucose and lipid metabolism (Supplementary material) (13, 19, 20). Cardiac imaging includes transthoracic echocardiography and coronary computed tomography angiography (CCTA) where clinically indicated. Continuous 72-h electrocardiogram (ECG) and heart rate variability (HRV) monitoring are performed at baseline using a clinically validated wearable device (ref) (Supplementary material). Data are collected using standardized procedures and processed according to predefined quality-control criteria to ensure comparability across study sites.

**Table 1:** Schedule of assessments and data collection in the AMI cohort study.

Evaluation parameters	Baseline	Follow-up							
		3 m	6 m	9 m	12 m	15 m	18 m	21 m	24 m
Informed consent	x								
Inclusion criteria met	x								
Standardized assessment*	x								
Sociodemographic characteristics+	x								
Lifestyle factors++	x								
Clinical history†	x								
Anthropometry and physiological parameters‡	x	x	x	x	x	x	x	x	x
Fasting venous blood test**	x	x	x	x	x	x	x	x	x
72-h wearable electrocardiogram monitoring¶	x		x		x		x		x
Transthoracic echocardiography¶¶	x				x				x
CCTA (if there are clinical indications)***	x				x				x
Assessment tool¶¶¶									
Rose Angina Questionnaire	x	x	x	x	x	x	x	x	x
Canadian Cardiovascular Society Angina Classification	x	x	x	x	x	x	x	x	x
PHQ-9	x	x	x	x	x	x	x	x	x
GAD-7	x	x	x	x	x	x	x	x	x
PSS-10	x	x	x	x	x	x	x	x	x
PSQI	x	x	x	x	x	x	x	x	x
FFQ	x	x	x	x	x	x	x	x	x
IPAQ-SF	x	x	x	x	x	x	x	x	x
GMAS	x	x	x	x	x	x	x	x	x

Baseline assessment: The project will be carried out from 2025 to 2027 in Gansu Provincial People’s Hospital, Aksay County People’s Hospital, Linxia Prefecture People’s Hospital, Gannan Tibetan Autonomous Prefecture People’s Hospital, and Dingxi City People’s Hospital. High-risk cardiovascular patients of the Han, Hui, Tibetan, and Kazakh ethnic groups will be identified through cardiovascular outpatient clinics, community screening programs, and electronic medical record systems. Follow-Up: it will be conducted at 3, 6, 9, 12, 15, 18, 21, and 24 months after enrollment in the cohort, for a period of 2 years. *Standardized assessment: Demographics, lifestyle, psychology, diet, and medication adherence questionnaires. +Sociodemographic characteristics: Age, sex, ethnicity, residence type, education, occupation, and income. ++Lifestyle factors: Smoking, alcohol consumption, dietary habits, physical activity, and sleep quality. †Clinical history: Hypertension, diabetes, dyslipidemia, previous stroke or TIA, family history of coronary heart disease, and history of drug use. ‡Anthropometry and physiological parameters: Including height, weight, BMI, waist circumference, blood pressure and body fat percentage. **Fasting venous blood tests: High-sensitivity C-reactive protein (hs-CRP), high-sensitivity troponin I (hs-TnI), creatine kinase (CK), creatine kinase-MB (CK-MB), lactate dehydrogenase (LDH), aspartate aminotransferase (AST), soluble suppression of tumorigenicity 2 (sST2), N-terminal pro–B-type natriuretic peptide (NT-proBNP), lipoprotein-associated phospholipase A2 (Lp-PLA2), fasting glucose, insulin, hemoglobin A1c (HbA1c), and a full lipid panel (total cholesterol, low-density lipoprotein cholesterol(LDL-C), high-density lipoprotein cholesterol (HDL-C), and triglycerides. ¶72-h wearable electrocardiogram (ECG) monitoring: A validated clinical-grade device (Shanghai Yishi Technology Co., Ltd.) will be used to continuously conduct 72-h wearable ECG and heart rate variability (HRV) monitoring. The data will be transmitted in real-time to a secure cloud platform, and the NARON CardioAI system will be used for automatic analysis, waveform classification, and feature extractio. ¶¶Transthoracic echocardiography: Unless there are other clinical indications, an echocardiogram will be performed once a year to evaluate the left ventricular ejection fraction and the situation of abnormal wall motion. ***Coronary computed tomography angiography (CCTA): If there are clinical indications should be repeated every 12 months. Assessment Tools: Includes the Rose Angina Questionnaire, The Canadian Cardiovascular Society Angina Classification, The Patient Health Questionnaire-9 (PHQ-9), The Generalized Anxiety Disorder-7 (GAD-7), The Perceived Stress Scale - 10 (PSS-10), The Pittsburgh Sleep Quality Index (PSQI), The Semi-quantitative Food Frequency Questionnaire (FFQ), The International Physical Activity Questionnaire Short Form (IPAQ-SF), The General Medication Adherence Scale (GMAS).

#### Follow-up assessments

Participants are followed every 3 months over 24 months using a standardized schedule (Table 1). This repeated-measures design allows assessment of temporal changes in risk indicators and their potential association with short-term cardiovascular events. At each visit, symptom status, interim medical events, medication use, and health-related behaviors are updated. Biomarkers are measured at regular intervals, and ECG monitoring is repeated every 6 months. Echocardiography is repeated annually, and CCTA is performed when clinically indicated. All suspected AMI events are adjudicated by an independent, blinded cardiology panel according to the Fourth Universal Definition of Myocardial Infarction (2018) (21).

#### Assessment tools

Standardized instruments are used to quantify lifestyle, psychological, and symptom-related variables (Supplementary Material 1). Angina symptoms are assessed using the Rose Angina Questionnaire and graded according to the Canadian Cardiovascular Society classification (22, 23). Psychological status is evaluated using the Patient Health Questionnaire-9 (PHQ-9) for depressive symptoms, the Generalized Anxiety Disorder-7 (GAD-7) scale for anxiety, and the Perceived Stress Scale-10 (PSS-10) for perceived stress (24–26). Sleep quality is assessed using the Pittsburgh Sleep Quality Index (PSQI) (27). Dietary intake is evaluated using a semi-quantitative food frequency questionnaire adapted to regional dietary patterns, and physical activity is assessed using the International Physical Activity Questionnaire Short Form (IPAQ-SF) (28, 29). Medication adherence is measured using the General Medication Adherence Scale (GMAS) (30).

#### Environmental and contextual data

Environmental variables, comprising monthly averaged meteorological parameters and air pollutant concentrations, were assigned to each patient according to their residential location and the date of hospital admission. Meteorological metrics, such as temperature, precipitation and relative humidity, were acquired from the ECMWF Reanalysis v5 (ERA5) product, which offers global coverage at a 0.25° × 0.25° gridded resolution (approximately 25 km at the equator) (3). For air pollution exposure assessment, concentrations of fine particulate matter (PM2.5 and PM10) (31), ozone (O3) (32), and nitrogen dioxide (NO₂) (33) were derived from the China High-Resolution and High-Quality Near-Surface Air Pollutants (CHAP) Dataset, which provides estimates at a high spatial resolution of 1 km. Elevation above sea level was also incorporated to account for its potential influence on AMI risk across different altitudes.

Healthcare access indicators include average travel time to the nearest secondary or tertiary hospital, hospital bed density, and physician density per 1,000 population (33). Information on socioeconomic factors, including city-level per capita income, employment patterns, occupational classifications, educational attainment, and housing conditions, will be systematically collected during scheduled follow-up visits and cross-referenced with official statistics from the Gansu Statistical Yearbook issued by the Gansu Provincial Bureau of Statistics, China.

#### Nested case–control component

Within the cohort, participants who develop incident AMI will be defined as cases, and matched controls will be selected from the risk set. Stored biospecimens from these participants will be used to assess variation in circulating biomarkers as short-term risk indicators prior to AMI, supporting analysis of temporal changes in risk within the cohort.

### Outcomes

The primary outcome is first incident AMI during the 24-month follow-up period. AMI events will be adjudicated according to the Fourth Universal Definition of Myocardial Infarction (2018), based on cardiac biomarker changes together with clinical, electrocardiographic, imaging, and symptom-related evidence. This outcome is defined to capture short-term occurrence of major cardiovascular events within individuals, allowing assessment of variation in near-term risk across populations with different environmental exposures, ethnic backgrounds, and healthcare accessibility.

Secondary outcomes include longitudinal changes in physiological and biochemical indicators, healthcare utilization, and short-term variation in risk markers preceding AMI. Repeated measurements of biomarkers and physiological parameters will be used to evaluate temporal changes within individuals, particularly within the 90 days preceding AMI, to characterize short-term fluctuations in cardiovascular risk. In addition, differences in these trajectories will be examined across environmental and ethnic subgroups to assess population-level heterogeneity in risk patterns.

#### Statistical analysis

All analyses will be conducted using R (version 4.3.0) and Python (version 3.10). Continuous variables will be summarized as mean (standard deviation) or median (interquartile range), and categorical variables as counts and percentages. Group comparisons will be performed using analysis of variance or Kruskal–Wallis tests for continuous variables, and chi-square tests for categorical variables. All statistical tests will be two-sided, with a significance threshold of *p* < 0.05. Adjustments for multiple comparisons will be applied as needed.

#### Primary outcome analysis

Associations between baseline variables and time to first incident AMI will be evaluated using Cox proportional hazards regression models. Follow-up time will be calculated from enrolment to AMI, loss to follow-up, death, or end of study. Multivariable models will include prespecified variables, including age, sex, ethnicity, smoking status, systolic blood pressure, diabetes status, lipid profile, and environmental exposures. Additional variables will be selected based on clinical relevance. Continuous variables will be modeled on their original scale, and nonlinearity will be assessed using restricted cubic splines. Hazard ratios (HRs) and 95% confidence intervals (CIs) will be reported. The proportional hazards assumption will be assessed using Schoenfeld residuals.

#### Longitudinal and short-term risk analysis

Repeated measurements of physiological and biomarker variables will be analyzed using linear mixed-effects models with random intercepts and random slopes when supported by the data. Time will be modeled as follow-up visit or continuous time since enrolment. Short-term risk will be evaluated using extended Cox models with time-varying covariates. Biomarker and physiological measurements will be updated at each follow-up visit and incorporated as time-dependent variables. For participants with incident AMI, measurements will be aligned relative to the event time. Predefined time windows preceding AMI will be constructed, and values within these windows will be analyzed. Within-individual changes will be defined as differences between consecutive measurements or relative to baseline values. Associations between time-updated variables and AMI risk will be estimated using time-varying Cox models.

#### Analysis of population heterogeneity

Subgroup analyses will be conducted across ethnicity, environmental exposure levels, and healthcare accessibility. Environmental exposures will be categorized into quantiles or clinically relevant categories. Interaction terms between key exposures and subgroup variables will be included in multivariable models. Stratified analyses will be performed when significant interactions are identified.

#### Risk prediction analysis

Prediction models incorporating baseline and time-varying variables will be developed. Candidate predictors will include demographic characteristics, clinical variables, environmental exposures, and repeated biomarker measurements. Predictor selection will be guided by clinical relevance and statistical criteria, including penalized regression methods. Model performance will be evaluated using discrimination and calibration. Discrimination will be assessed using the concordance index (C-statistic), and calibration will be assessed using calibration plots and calibration slope. Internal validation will be conducted using bootstrap resampling.

#### Additional analyses

Within the nested case–control component, conditional logistic regression models will be used to assess associations between biomarker indicators and AMI risk. Exploratory analyses using machine learning methods will be conducted to assess nonlinear relationships and interactions. Sensitivity analyses will be performed to evaluate the robustness of findings. Missing data will be addressed using multiple imputation.

## Discussion

AMI remains a leading cause of mortality worldwide, and its burden continues to be substantial in China (1, 2). Conventional tools such as the Framingham Risk Score and China-PAR are designed to estimate long-term cardiovascular risk based on baseline characteristics and are widely used in population screening (11, 13). However, these models assume relative stability of risk profiles and do not capture short-term variation preceding acute events. This limitation is particularly relevant for first incident AMI, which represents the transition from a high-risk state to overt disease before treatment and secondary prevention modify the clinical course. Recent work has highlighted the value of dynamic and time-updated cardiovascular risk assessment (14), yet evidence remains limited regarding whether short-term risk trajectories preceding first AMI differ across populations exposed to different environmental and ethnic contexts. The present study addresses this gap within a multiethnic and environmentally heterogeneous setting. Repeated assessments of behavioral, psychosocial, physiological, and biomarker indicators capture changes preceding first AMI that are not reflected by baseline risk profiles alone.

A central contribution of this study is the integration of multidimensional risk indicators, with particular emphasis on behavioral, psychosocial, and lifestyle domains. Repeated assessments of physical activity, sleep quality, perceived stress, anxiety, depression, diet, and medication adherence provide a structured representation of functional health status and its variation over time. The selected instruments, including PHQ-9, GAD-7, PSS-10, PSQI, IPAQ-SF, FFQ, and GMAS, have been validated in previous studies (24–29, 34). These dimensions capture aspects of cardiovascular vulnerability that are directly relevant to public health, as they reflect modifiable pathways linking behavioral and social conditions to disease risk (5, 6). Importantly, longitudinal assessment of these indicators enables identification of short-term changes preceding first AMI. Within this framework, biological and physiological measurements are incorporated as complementary components to reflect underlying variation across individuals. Prior studies have shown that biomarkers and physiological indicators, including heart rate variability, are associated with cardiovascular risk stratification (35–42). Together, these domains support a multidimensional framework in which short-term cardiovascular risk is embedded in behavioral, psychosocial, and biological processes. First AMI does not necessarily occur on a stable background of risk factors. Changes across these domains may accompany the transition from high-risk status to overt disease. Short-term risk may therefore depend not only on risk level but also on changes over time.

The study is particularly distinctive in its explicit focus on environmental and ethnic differences as sources of heterogeneity in short-term risk. Cardiovascular risk is influenced by geographic context, healthcare accessibility, and social conditions (2, 4). In China, ethnic disparities in cardiovascular risk factors and disease burden have been widely documented and are associated with differences in lifestyle, diet, and social determinants of health (5, 6) Kazakh populations have distinct cardiometabolic profiles (7, 8), and studies in Hui populations have reported differences in health behaviour and information-seeking patterns (42). Tibetan populations living at high altitude exhibit physiological characteristics related to hypoxic adaptation (10), and recent studies have described population-specific adaptation mechanisms (32). These ethnic differences coexist with environmental heterogeneity, including variation in altitude, air pollution, and healthcare accessibility. Environmental exposures such as particulate matter and meteorological variation have been associated with cardiovascular outcomes (3), and high-resolution datasets enable spatial characterization of environmental exposures and healthcare accessibility across China (32, 33). These population and environmental differences suggest that similar changes in symptoms, behaviors, physiological measurements, or biomarkers may not correspond to the same degree of short-term risk. Interpretation of changes before first AMI may therefore depend on the environmental and population context in which they occur. Future findings from this study may indicate whether risk progression before first AMI follows common patterns or differs across populations.

Several limitations should be acknowledged. The hospital-based design may limit generalizability to populations with lower healthcare access. Residual confounding may persist, particularly for social and environmental factors that are not fully captured by the variables collected in this study. Although repeated measurements allow assessment of temporal changes, the follow-up interval may not fully capture very rapid fluctuations immediately preceding an event. Behavioral and psychosocial measures may be affected by reporting bias despite the use of validated instruments. Tobacco exposure is characterized by smoking status only, and specific tobacco product types are not separately recorded. Consequently, the effects of different forms of tobacco use cannot be distinguished. The nested case–control component should be interpreted within the broader cohort framework and primarily supports efficient evaluation of pre-event variation (43, 44).

Despite these limitations, the study has important strengths, including its focus on first incident AMI, its use of repeated multidimensional assessments, and its inclusion of diverse populations across distinct environmental settings. These features provide a basis for examining how short-term cardiovascular risk varies across environmental and ethnic contexts. Conventional risk scores identify individuals with elevated risk but provide limited information on when risk begins to change. Repeated measurements before first AMI address this question directly. If short-term changes contribute little beyond baseline factors, such findings would support refinement rather than replacement of current risk assessment strategies.

## Conclusion

In conclusion, short-term risk preceding first incident AMI varies across environmental and ethnic contexts. By integrating repeated behavioral, psychosocial, and contextual data within a longitudinal framework, this study conceptualizes cardiovascular risk as a dynamic process shaped by real-life conditions. These findings support the development of population-specific and context-sensitive strategies for early identification and prevention of AMI.

### Platform and data storage

All study data will be collected using standardized procedures and entered into a secure, password-protected database. The dataset will be maintained on a dedicated server within the Specific-Disease Database of Gansu Provincial Hospital, with access limited to authorized research personnel. Data management procedures will be implemented to ensure data integrity, confidentiality, and compliance with relevant data protection standards.
