# Supplementary material for: Environmental and ethnic differences in short-term risk of first acute myocardial infarction: a prospective multiethnic cohort protocol
Source: Front Public Health. 2026 Aug 12;14:1848166. doi: 10.3389/fpubh.2026.1848166 (PMC13506950; doi:10.3389/fpubh.2026.1848166)
Supplement: Supplementary file 1 [file Table_1.DOCX]

Supplementary Material 1. Detailed variable definitions, measurement procedures, and data processing

1. Biomarker measurements

Venous blood samples are collected after an overnight fast of at least 8 hours at each scheduled visit. Samples are processed and analyzed in certified clinical laboratories according to standardized protocols.

Inflammatory and cardiac biomarkers include high-sensitivity C-reactive protein (hs-CRP, mg/L), high-sensitivity cardiac troponin I (hs-TnI, ng/L), soluble suppression of tumorigenicity 2 (sST2, ng/mL), N-terminal pro–B-type natriuretic peptide (NT-proBNP, pg/mL), and lipoprotein-associated phospholipase A2 (Lp-PLA2, ng/mL).

Cardiac enzymes include creatine kinase (CK, U/L), creatine kinase-MB (CK-MB, U/L), lactate dehydrogenase (LDH, U/L), and aspartate aminotransferase (AST, U/L).

Metabolic indicators include fasting plasma glucose (FPG, mmol/L), fasting insulin (μIU/mL), glycated hemoglobin (HbA1c, %), total cholesterol (TC, mmol/L), low-density lipoprotein cholesterol (LDL-C, mmol/L), high-density lipoprotein cholesterol (HDL-C, mmol/L), and triglycerides (TG, mmol/L).

All assays are performed using standardized commercial kits and automated analyzers. Internal quality control is conducted regularly, and external quality assessment is performed according to national laboratory standards.

2. Electrocardiogram (ECG) and heart rate variability (HRV) analysis

Continuous electrocardiographic monitoring is performed over 72 hours using a validated wearable device. Recordings are obtained under free-living conditions.

ECG features extracted include P-wave duration (ms), QRS complex duration (ms), and T-wave amplitude (mV).

HRV metrics are calculated using standard time-domain and frequency-domain methods, including:

- Standard deviation of normal-to-normal intervals (SDNN, ms)

- Root mean square of successive differences (RMSSD, ms)

- Low-frequency power (LF, ms²)

- High-frequency power (HF, ms²)

- LF/HF ratio

Signal preprocessing includes artifact detection, noise filtering, and exclusion of non-sinus beats. Recordings with less than 80% valid data or insufficient wear time are excluded from analysis.

3. Wearable device and data acquisition

Physiological data are collected using a clinically validated wearable electrocardiographic monitoring device. Data are continuously recorded during the monitoring period and stored in a secure digital platform.

Signal processing is performed using standardized algorithms, including noise reduction, beat detection, and feature extraction. All procedures are conducted using predefined pipelines to ensure consistency across study sites.

4. Environmental exposure assessment

Environmental exposure data are assigned based on participants’ residential addresses and follow-up periods.

Meteorological variables, including temperature, precipitation, and relative humidity, are obtained from the ECMWF Reanalysis v5 (ERA5) dataset, with a spatial resolution of 0.25° × 0.25°.

Air pollution data, including fine particulate matter (PM2.5 and PM10), ozone (O₃), and nitrogen dioxide (NO₂), are derived from the China High-Resolution and High-Quality Near-Surface Air Pollutants (CHAP) dataset, with a spatial resolution of 1 km.

Exposure levels are matched to individual participants based on residential location and time of follow-up. Monthly averages are calculated to represent short-term environmental exposure.

Altitude is determined using geospatial elevation data and incorporated as a geographic exposure variable.

5. Healthcare access and socioeconomic indicators

Healthcare accessibility is assessed using indicators including travel time to the nearest secondary or tertiary hospital, hospital bed density, and physician density per 1,000 population.

Socioeconomic variables include education level, occupation, income, and housing conditions, collected through standardized questionnaires.

Regional-level socioeconomic data are obtained from official statistics, including the Gansu Statistical Yearbook. These variables are used to characterize contextual differences across study regions.

6. Data quality control

Standardized operating procedures are implemented across all study sites for data collection and processing.

Quality control measures include:

1) Training and certification of study personnel
2) Calibration of measurement devices
3) Regular monitoring of data completeness and consistency
4) Automated checks for outliers and implausible values

Missing data are documented and handled using predefined statistical approaches. Sensitivity analyses will be conducted to assess the impact of missing data on study findings.
