# Supplementary material for: Environmental and ethnic differences in short-term risk of first acute myocardial infarction: a prospective multiethnic cohort protocol
Source: Front Public Health. 2026 Aug 12;14:1848166. doi: 10.3389/fpubh.2026.1848166 (PMC13506950; doi:10.3389/fpubh.2026.1848166)
Supplement: Supplementary file 2 [file Data_Sheet_1.ZIP › 评分/Assessment tools(1)(1).pdf]

The Rose Angina Questionnaire

Introduction: I am going to ask you a few questions about pain or discomfort you might feel in your chest. This helps us understand your health better.

Part 1

1.Do you ever have any pain or discomfort in your chest?

☐No → (If No, stop the questionnaire. The subject is classified as having no angina.)

☐Yes

Part 2 (Only if answer to Question 1 is "Yes")

2.Do you get this pain or discomfort when you walk uphill or hurry?

☐No

☐Yes

3.Do you get it when you walk at an ordinary pace on the level?

☐No

☐Yes

If "Yes" to Question 2 or 3, continue. If "No" to both, stop. No angina.

4.What do you do if you get it while you are walking?

☐Stop or slow down

☐Continue at the same pace

5.What happens to it if you stand still?

☐The pain usually goes away

☐The pain does not go away until you do something else (e.g., take medicine)

For a positive response: The subject must "Stop or slow down" (Q4) and the pain must "Go away" (Q5).

Part 3 (Only if criteria from Part 2 are met)

6.Where do you feel the pain?

(Show the subject a diagram pointing to the sternum and the left arm.)

☐The pain is located in the sternum or in the left arm and chest.

☐The pain is located elsewhere.

For a diagnosis of "definite angina," the pain must be in the sternum or left arm/chest.

Scoring and Interpretation
The questionnaire is designed to be interpreted in a specific sequence to classify individuals.

Classification Criteria Met
Definite Angina Pectoris
-Yes to Question 1
-Yes to Question 2 or Question 3
- "Stop or slow down" on Question 4
-"The pain usually goes away" on Question 5
-Sternum or left arm/chest location on Question 6
No Angina
-No to Question 1, OR No to both Questions 2 and 3, OR Fails to meet the criteria for "stopping" and "pain going away" (Q4 & Q5).

Additional Notes (Recommended for a comprehensive assessment)
Have you ever had a severe pain across the front of your chest lasting for half an hour or more?

☐No

☐Yes

This question aims to identify possible myocardial infarction (heart attack) history. A "Yes" answer would warrant further investigation but is not part of the original angina classification.

Canadian Cardiovascular Society (CCS) Classification of Angina

Class I

☐"Ordinary physical activity does not cause angina."

☐Angina does not occur with walking or climbing stairs at a normal pace.

☐Angina occurs with strenuous, rapid, or prolonged exertion at work or recreation.

☐Patients are not limited in their ordinary activities.

Class II

☐"Slight limitation of ordinary activity."

☐Angina occurs with:

☐Walking or climbing stairs rapidly.

☐Walking uphill.

☐Walking or climbing stairs after meals, in cold, in wind, or under emotional stress.

☐Walking more than two blocks on level ground.

☐Climbing more than one flight of stairs at a normal pace.

☐Patients are mildly limited. They can perform most daily activities, but these specific factors will provoke symptoms.

Class III

☐"Marked limitation of ordinary physical activity."

☐Angina occurs with:

☐Walking one to two blocks on level ground.

☐Climbing one flight of stairs at a normal pace and under normal conditions.

☐ Patients are significantly limited. They are comfortable at rest, but even light daily tasks will bring on angina.

Class IV

☐"Inability to carry on any physical activity without discomfort."

☐Angina symptoms may be present at rest.

☐Any physical activity invariably precipitates angina.

☐Patients are severely limited and cannot perform most activities without discomfort.

Patient Health Questionnaire (PHQ-9)

Over the last two weeks, how often have you been bothered by any of the following problems?

	**Not At All**	**Several days**	**More than half the days**	**Nearly everyday**
1. Little interest or pleasure in doing things	0	1	2	3
2. Feeling down, depressed, or hopeless	0	1	2	3
**Scores (add columns)**	__________ +	______________ +	______________ +	____________
**Front Total Score**				

Ifyour total score is greater than or equal to three,please continue with questions 3-10 on the back.

Over the last two weeks, how often have you been bothered by any of the following problems?

	Not at all	Several days	More than half the days	Nearly everyday
3.Trouble falling or staying asleep, or sleeping too much	0	1	2	3
4. Feeling tired of having little energy	0	1	2	3
5. Poor appetite or overeating	0	1	2	3
6. Feeling bad about yourself -- or that you are a failure or having let yourself or your family down	0	1	2	3
7. Trouble concentrating on things, such as reading the newspaper or watching television	0	1	2	3
8. Moving or speaking so slowly that other people could have noticed. Or the opposite -- being so fidgety or restless that you have been moving a lot more than usual	0	1	2	3
9. Thoughts that you would be better off dead, or of hurting yourself in some way	0	1	2	3
**Scores (add columns)**	____+________+____________+_________			
**Back Total Score**				

10. If you circle any problems, how difficult have these problems made it for you to do your work, study, go to class or get alone with other people?	**Not difficult at all** ☐
	**Somewhat difficult** ☐
	**Very difficult** ☐
	**Extremely difficult** ☐

Total Score from Front	Total Score form Back	Grant Total

Generalized Anxiety Disorder-7(GAD-7)

	Have you often been troubled by the following problems in the past two weeks? (Please tick your answer with "√".)	Not At All	Several days	More than half the days	Nearly everyday
1	Feeling restless, anxious or highly tense.	0	1	2	3
2	Can't stop or control worrying.	0	1	2	3
3	Worry excessively about all sorts of things.	0	1	2	3
4	It's difficult to relax.	0	1	2	3
5	It's so noisy that it's hard to sit still.	0	1	2	3
6	Become irritable or impatient.	0	1	2	3
7	Be afraid of terrible things happening.	0	1	2	3

(For office coding: Total Score T _____ = _____ + _____ + _____)

Perceived Stress Scale - 10 (PSS - 10)

1.In the past month, how many times have you felt nervous or "stressed out"?
A. Never
B. Almost never (less than once)
C. Sometimes (1 - 2 times)
D. Often (3 - 4 times)
E. Always (5 times or more)
2.In the past month, how many times have you felt confident that you could handle your personal problems? (Reverse scoring question)
A. Never
B. Almost never (less than once)
C. Sometimes (1 - 2 times)
D. Often (3 - 4 times)
E. Always (5 times or more)
3.In the past month, how many times have you felt that things were going as you wished? (Reverse scoring question)
A. Never
B. Almost never (less than once)
C. Sometimes (1 - 2 times)
D. Often (3 - 4 times)
E. Always (5 times or more)
4.In the past month, how many times have you found that you couldn't control the important things in your life?
A. Never
B. Almost never (less than once)
C. Sometimes (1 - 2 times)
D. Often (3 - 4 times)
E. Always (5 times or more)
5.In the past month, how many times have you felt angry because things were going beyond your expectations?
A. Never
B. Almost never (less than once)
C. Sometimes (1 - 2 times)
D. Often (3 - 4 times)
E. Always (5 times or more)
6.In the past month, how many times have you felt that difficulties were piling up and you couldn't overcome them?
A. Never
B. Almost never (less than once)
C. Sometimes (1 - 2 times)
D. Often (3 - 4 times)
E. Always (5 times or more)
7.In the past month, how many times have you felt happy? (Reverse scoring question)
A. Never
B. Almost never (less than once)
C. Sometimes (1 - 2 times)
D. Often (3 - 4 times)
E. Always (5 times or more)

8.In the past month, how many times have you felt that you were effective and capable? (Reverse scoring question)
A. Never
B. Almost never (less than once)
C. Sometimes (1 - 2 times)
D. Often (3 - 4 times)
E. Always (5 times or more)
9.In the past month, how many times have you felt that you could control what was happening in your life? (Reverse scoring question)
A. Never
B. Almost never (less than once)
C. Sometimes (1 - 2 times)
D. Often (3 - 4 times)
E. Always (5 times or more)
10.In the past month, how many times have you felt overwhelmed by the things you had to do?
A. Never
B. Almost never (less than once)
C. Sometimes (1 - 2 times)
D. Often (3 - 4 times)
E. Always (5 times or more)

PSS - 10 Scoring Algorithm

For positively - scored questions (such as Questions 1, 4, 5, 6, and 10), choose Option A to get 0 points, Option B to get 1 point, Option C to get 2 points, Option D to get 3 points, and Option E to get 4 points.

For negatively - scored questions (such as Questions 2, 3, 7, 8, and 9), choose Option A to get 4 points, Option B to get 3 points, Option C to get 2 points, Option D to get 1 point, and Option E to get 0 points. Calculate the total score of the 10 questions. The total score ranges from 0 to 40 points. The higher the score, the greater the perceived stress.

Pittsburgh Sleep Quality Index (PSQI)

	**Project**	**Score**			
		**0**	**1**	**2**	**3**
1	In the past month, I usually go to bed at o'clock at night.				
2	In the past month, it usually takes ____ minutes to fall asleep after getting into bed.	□≤15min	□16~30min	□31~60min	□≥60min
3	In the past month, usually get up at ____ o'clock in the morning.				
4	In the past month, time spent in bed is ____hours (not equal to the time in bed)				
5	In the past month, I've been troubled by sleep problems caused by the following situations.				
	a. Difficulty falling asleep (unable to fall asleep within 30 minutes)	□None	□ < 1 time a week	□ 1 - 2 times a week	□ ≥ 3 times a week
	b. Easily wake up at night or wake up early.	□None	□ < 1 time a week	□ 1 - 2 times a week	□ ≥ 3 times a week
	c. Go to the toilet at night.	□None	□ < 1 time a week	□ 1 - 2 times a week	□ ≥ 3 times a week
	d. Difficulty breathing	□None	□ < 1 time a week	□ 1 - 2 times a week	□ ≥ 3 times a week
	e. Loud coughing or snoring	□None	□ < 1 time a week	□ 1 - 2 times a week	□ ≥ 3 times a week
	f. feel cold.	□None	□ < 1 time a week	□ 1 - 2 times a week	□ ≥ 3 times a week
	g. Feel hot	□None	□ < 1 time a week	□ 1 - 2 times a week	□ ≥ 3 times a week
	h. Have a nightmare.	□None	□ < 1 time a week	□ 1 - 2 times a week	□ ≥ 3 times a week
	i. Pain and discomfort	□None	□ < 1 time a week	□ 1 - 2 times a week	□ ≥ 3 times a week
	j. Other things that affect sleep If yes, please specify:	□None	□ < 1 time a week	□ 1 - 2 times a week	□ ≥ 3 times a week
6	In the past month, generally speaking, what do you think of your sleep quality?	□Very good	□Relatively good	□Poor	□Very poor
7	In the past month, how have you used medication for hypnosis?	□None	□ < 1 time a week	□ 1 - 2 times a week	□ ≥ 3 times a week
8	Have you often felt sleepy in the past month?	□None	□ < 1 time a week	□ 1 - 2 times a week	□ ≥ 3 times a week
9	Have you lacked the energy to do things in the past month?	□None	□Occasionally	□Sometimes there is	□Often have

Scoring method:

constituent	content	Score			
		0	1	2	3
A. Sleep quality	Item 6 Scoring	□Very good	□Relatively good	□Poor	□Very poor
B. Bedtime	Scoring cumulative for Items 2 and 5a	□ 0 points	□ 1 - 2 points	□ 3 - 4 points	□ 5 - 6 points
C. Sleep time	Item 4 Scoring	□ > 7 hours	□ 6 - 7 hours (excluding 6 hours)	□ 5 - 6 hours (including 6 hours)	□ < 5 hours
D. Sleep efficiency	Calculate sleep efficiency based on the responses to items 1, 3, and 4.	□ > 85%	□ 75 - 85% (excluding 75%)	□ 65 - 75% (including 75%)	□ < 65%
E. Sleep disorders	Scoring cumulative for items 5b - 5j	□ 0 points	□ 1 - 9 points	□ 10 - 18 points	□ 19 - 27 points
F、Hypnotic drugs	Item 7 Scoring	□None	□ < 1 time a week	□ 1 - 2 times a week	□ ≥ 3 times a week
G. Daytime dysfunction	Scoring cumulative of items 8 and 9	□ 0 points	□ 1 - 2 points	□ 3 - 4 points	□ 5 - 6 points

Sleep efficiency calculation method:

Sleep efficiency =Item 4 (Sleep time)/(Item 3 (Wake-up time)-Item 1 (Bedtime))*100%

International Physical Activity Questionnaire Short Form (IPAQ-SF)

PART 1: VIGOROUS-INTENSITY ACTIVITIES

Vigorous activities refer to activities that take hard physical effort and make you breathe much harder than normal. Think only about those physical activities that you did for at least 10 minutes at a time.

Examples: heavy lifting, digging, aerobics, fast cycling, or competitive sports (e.g., basketball, soccer, singles tennis).

During the last 7 days, on how many days did you do vigorous physical activities?

____ days per week

If your answer is zero, please skip to Question 3 (about walking).

How much time did you usually spend on one of those days doing vigorous physical activities?

____ hours per day

____ minutes per day

PART 2: MODERATE-INTENSITY ACTIVITIES

Moderate activities refer to activities that take moderate physical effort and make you breathe somewhat harder than normal.

Examples: carrying light loads, bicycling at a regular pace, doubles tennis. Do not include walking.

During the last 7 days, on how many days did you do moderate physical activities?

____ days per week

If your answer is zero, please skip to Question 5 (about sitting).

How much time did you usually spend on one of those days doing moderate physical activities?

____ hours per day

____ minutes per day

PART 3: WALKING
During the last 7 days, on how many days did you walk for at least 10 minutes at a time?

____ days per week

If your answer is zero, please skip to Question 7 (about sitting).

How much time did you usually spend on one of those days walking?

____ hours per day

____ minutes per day

PART 4: SITTING
This question is about the time you spent sitting while at work, at home, while doing coursework, and during leisure time (e.g., sitting at a desk, visiting friends, reading, or watching television). Do not include time spent sitting in a vehicle.

During the last 7 days, how much time did you spend sitting on a weekday?

____ hours per day

____ minutes per day

Thank you for your participation.

IPAQ-SF SCORING PROTOCOL
Important Note: This is a simplified overview. For formal research, please consult the official IPAQ scoring protocol.

Convert to MET-minutes/week:

MET (Metabolic Equivalent of Task) is a unit used to estimate the energy cost of activities.

Vigorous activities = 8.0 METs

Moderate activities = 4.0 METs

Walking = 3.3 METs

Calculation Formula:

Vigorous MET-min/week = (Days of vigorous activity) × (Minutes per day) × 8.0

Moderate MET-min/week = (Days of moderate activity) × (Minutes per day) × 4.0

Walking MET-min/week = (Days of walking) × (Minutes per day) × 3.3

Total Physical Activity:

Total MET-min/week = Sum of Vigorous, Moderate, and Walking MET-minutes/week.

Categorizing Physical Activity Levels:

High:

Vigorous activity on at least 3 days achieving a minimum of 1500 MET-min/week OR Any combination of walking, moderate, or vigorous activities on at least 7 days achieving a minimum of 3000 MET-min/week.

Moderate:

At least 20 minutes of vigorous activity on at least 3 days OR At least 30 minutes of moderate activity or walking on at least 5 days OR Any combination achieving a minimum of 600 MET-min/week.

Low: The lowest level of activity. Individuals not meeting the criteria for "Moderate" or "High" are categorized here.

Sitting Time:

Reported as the average minutes (or hours) spent sitting on a weekday. This is analyzed separately.

General Medication Adherence Scale（GMAS）

Item	Content
Patient	______________

Questionnaire Items

GMAS 1: General Medication Adherence

No.	Question	0 Never	1 Rarely	2 Sometimes	3 Often
1	Do you have difficulty remembering to take your medication regularly?	☐	☐	☐	☐
2	Have you forgotten to take medication due to busy life (work, travel, parties, weddings, festivals)?	☐	☐	☐	☐
3	Do you stop medication when you feel better?	☐	☐	☐	☐
4	Do you stop medication due to side effects (stomach pain, nausea, digestive issues)?	☐	☐	☐	☐
5	Do you stop medication without informing your doctor?	☐	☐	☐	☐

GMAS 2: Comorbidity & Concurrent Medication

No.	Question	0 Never	1 Rarely	2 Sometimes	3 Often
6	Do you skip current medication because of other medications?	☐	☐	☐	☐
7	Are you unable to get medication due to expired prescription?	☐	☐	☐	☐
8	Do you forget old medication when new medications are added?	☐	☐	☐	☐
9	Do you adjust dose/timing without doctor’s advice?	☐	☐	☐	☐

GMAS 3: Economic & Perceived Effectiveness

No.	Question	0 Never	1 Rarely	2 Sometimes	3 Often
10	Are you unable to buy medicine due to financial problems?	☐	☐	☐	☐
11	Do you think the medicine is not effective?	☐	☐	☐	☐

3. Scoring System

Domain Scores & Grading

Domain	Score Range	Adherence Grade
GMAS 1	13 – 15	High
	11 – 12	Good
	8 – 10	Partial
	5 – 7	Low
	0 – 4	Poor
GMAS 2	11 – 12	High
	9 – 10	Good
	6 – 8	Partial
	4 – 5	Low
	0 – 3	Poor
GMAS 3	6	High
	5	Good

Domain	Score Range	Adherence Grade
	3 – 4	Partial
	2	Low
	0 – 1	Poor

Overall Adherence (Total Score)

Total Score	Adherence Level
30 – 33	High Adherence
27 – 29	Good Adherence
17 – 26	Partial Adherence
11 – 16	Low Adherence
0 – 10	Poor Adherence

4.Scoring Sheet (for researchers)

Domain	Total Score	Grade
GMAS 1	________	________
GMAS 2	________	________
GMAS 3	________	________
**Overall Total**	________	________
