# Supplementary material for: Environmental and ethnic differences in short-term risk of first acute myocardial infarction: a prospective multiethnic cohort protocol
Source: Front Public Health. 2026 Aug 12;14:1848166. doi: 10.3389/fpubh.2026.1848166 (PMC13506950; doi:10.3389/fpubh.2026.1848166)
Supplement: Supplementary file 2 [file Data_Sheet_1.ZIP › 评分/FFQ(1).pdf]

NHANES Food Questionnaire

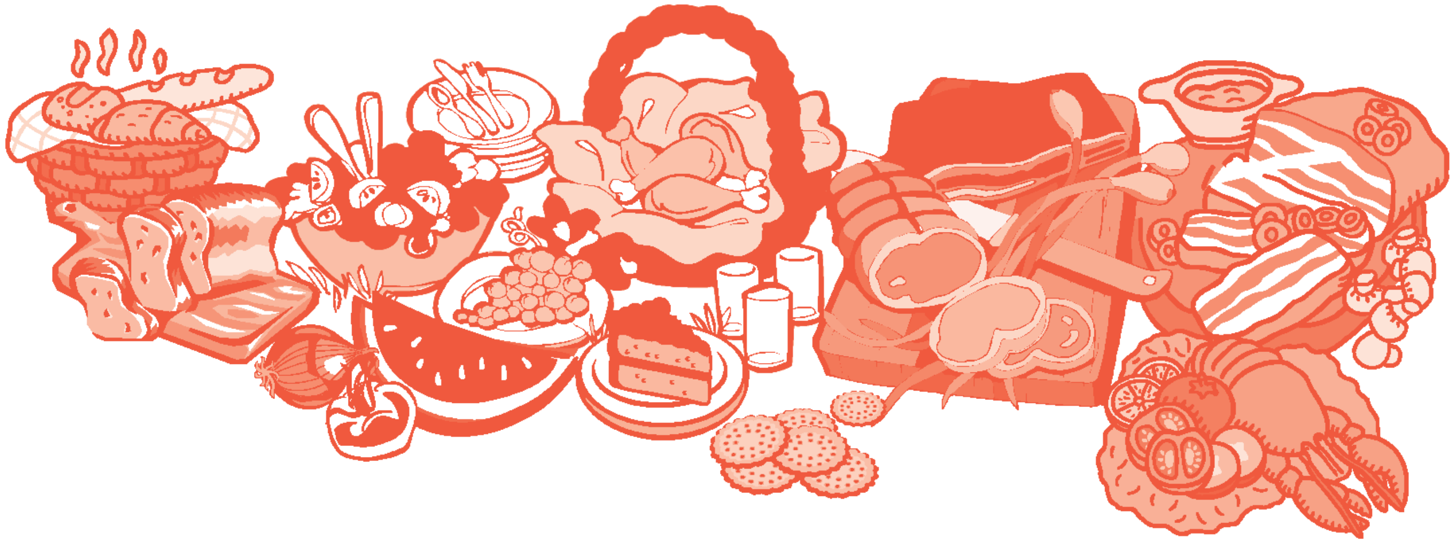

More than one member of your household may have received a questionnaire. Please make sure this is your booklet before answering any questions.

LABEL HERE

3/8" SPINE PERF

GENERAL INSTRUCTIONS

- **Answer each question as best you can. Estimate if you are not sure. A guess is better than leaving a blank.**
- **Use only a No. 2 pencil.**
- **Be certain to completely blacken in each of the answers.**
- **Erase completely if you make any changes.**
- **Do not make any stray marks on this form.**
- **If you blacken NEVER or NO for a question, please follow any arrows or instructions that direct you to the next question.**

PLEASE DO NOT WRITE IN THIS AREA

SERIAL #

244004 - 1/6

1. Over the past 12 months, how often did you drink **tomato juice** or **vegetable juice**?

- ◯ NEVER

- ◯ 1 time per month or less
- ◯ 2–3 times per month
- ◯ 1–2 times per week
- ◯ 3–4 times per week
- ◯ 5–6 times per week
- ◯ 1 time per day
- ◯ 2–3 times per day
- ◯ 4–5 times per day
- ◯ 6 or more times per day

2. How often did you drink **orange juice** or **grapefruit juice?**

- ◯ NEVER

- ◯ 1 time per month or less
- ◯ 2–3 times per month
- ◯ 1–2 times per week
- ◯ 3–4 times per week
- ◯ 5–6 times per week
- ◯ 1 time per day
- ◯ 2–3 times per day
- ◯ 4–5 times per day
- ◯ 6 or more times per day

3. How often did you drink **apple juice?**

- ◯ NEVER

- ◯ 1 time per month or less
- ◯ 2–3 times per month
- ◯ 1–2 times per week
- ◯ 3–4 times per week
- ◯ 5–6 times per week
- ◯ 1 time per day
- ◯ 2–3 times per day
- ◯ 4–5 times per day
- ◯ 6 or more times per day

4. How often did you drink **grape juice?**

- ◯ NEVER

- ◯ 1 time per month or less
- ◯ 2–3 times per month
- ◯ 1–2 times per week
- ◯ 3–4 times per week
- ◯ 5–6 times per week
- ◯ 1 time per day
- ◯ 2–3 times per day
- ◯ 4–5 times per day
- ◯ 6 or more times per day

5. How often did you drink **other 100% fruit juice** or **100% fruit juice mixtures** (such as pineapple, prune, or others)?

- ◯ NEVER

- ◯ 1 time per month or less
- ◯ 2–3 times per month
- ◯ 1–2 times per week
- ◯ 3–4 times per week
- ◯ 5–6 times per week
- ◯ 1 time per day
- ◯ 2–3 times per day
- ◯ 4–5 times per day
- ◯ 6 or more times per day

6. How often did you drink other **fruit drinks** (such as cranberry cocktail, Hi-C, lemonade, or Kool-Aid, diet or regular)?

- ◯ NEVER **(GO TO QUESTION 7)**

- ◯ 1 time per month or less
- ◯ 2–3 times per month
- ◯ 1–2 times per week
- ◯ 3–4 times per week
- ◯ 5–6 times per week
- ◯ 1 time per day
- ◯ 2–3 times per day
- ◯ 4–5 times per day
- ◯ 6 or more times per day

6a. How often were your fruit drinks **diet** or **sugar-free drinks**?

- ◯ Almost never or never
- ◯ About $^1/_4$ of the time
- ◯ About $^1/_2$ of the time
- ◯ About $^3/_4$ of the time
- ◯ Almost always or always

7. How often did you drink **milk as a beverage** (NOT in coffee, NOT in cereal)? (Please include chocolate milk and hot chocolate.)

- ◯ NEVER **(GO TO QUESTION 8)**

- ◯ 1 time per month or less
- ◯ 2–3 times per month
- ◯ 1–2 times per week
- ◯ 3–4 times per week
- ◯ 5–6 times per week
- ◯ 1 time per day
- ◯ 2–3 times per day
- ◯ 4–5 times per day
- ◯ 6 or more times per day

7a. What kind of **milk** did you usually drink?

- ◯ Whole milk
- ◯ 2% fat milk
- ◯ 1% fat milk
- ◯ Skim, nonfat, or $^1/_2$% fat milk
- ◯ Soy milk
- ◯ Rice milk
- ◯ Raw, unpasteurized milk
- ◯ Other

BAR
CODE
LABEL
HERE

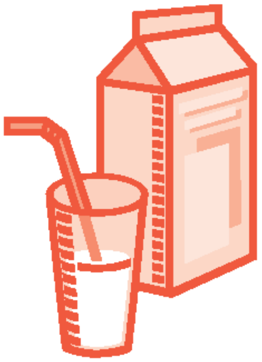

Question 8 appears on the next page.

3/8" SPINE PERF

Over the past 12 months...

8. How often did you drink **meal replacement, energy, or high-protein beverages** such as Instant Breakfast, Ensure, Slimfast, Sustacal or others?

◯ NEVER

◯ 1 time per month or less
◯ 2–3 times per month
◯ 1–2 times per week
◯ 3–4 times per week
◯ 5–6 times per week
◯ 1 time per day
◯ 2–3 times per day
◯ 4–5 times per day
◯ 6 or more times per day

9. Over the past 12 months, did you drink **soft drinks, soda,** or **pop**?

◯ NO **(GO TO QUESTION 10)**

◯ YES

9a. How often did you drink **soft drinks, soda,** or **pop IN THE SUMMER**?

◯ NEVER

◯ 1 time per month or less
◯ 2–3 times per month
◯ 1–2 times per week
◯ 3–4 times per week
◯ 5–6 times per week
◯ 1 time per day
◯ 2–3 times per day
◯ 4–5 times per day
◯ 6 or more times per day

9b. How often did you drink **soft drinks, soda,** or **pop DURING THE REST OF THE YEAR**?

◯ NEVER

◯ 1 time per month or less
◯ 2–3 times per month
◯ 1–2 times per week
◯ 3–4 times per week
◯ 5–6 times per week
◯ 1 time per day
◯ 2–3 times per day
◯ 4–5 times per day
◯ 6 or more times per day

9c. How often were these soft drinks, soda, or pop **diet** or **sugar-free**?

◯ Almost never or never
◯ About 1/4 of the time
◯ About 1/2 of the time
◯ About 3/4 of the time
◯ Almost always or always

9d. How often were these soft drinks, soda, or pop **caffeine-free**?

◯ Almost never or never
◯ About 1/4 of the time
◯ About 1/2 of the time
◯ About 3/4 of the time
◯ Almost always or always

Question 10 appears in the next column.

10. Over the past 12 months, did you drink **beer**?

◯ NO **(GO TO QUESTION 11)**

◯ YES

10a. How often did you drink **beer IN THE SUMMER**?

◯ NEVER

◯ 1 time per month or less
◯ 2–3 times per month
◯ 1–2 times per week
◯ 3–4 times per week
◯ 5–6 times per week
◯ 1 time per day
◯ 2–3 times per day
◯ 4–5 times per day
◯ 6 or more times per day

10b. How often did you drink **beer DURING THE REST OF THE YEAR**?

◯ NEVER

◯ 1 time per month or less
◯ 2–3 times per month
◯ 1–2 times per week
◯ 3–4 times per week
◯ 5–6 times per week
◯ 1 time per day
◯ 2–3 times per day
◯ 4–5 times per day
◯ 6 or more times per day

11. How often did you drink **wine** or **wine coolers**?

◯ NEVER

◯ 1 time per month or less
◯ 2–3 times per month
◯ 1–2 times per week
◯ 3–4 times per week
◯ 5–6 times per week
◯ 1 time per day
◯ 2–3 times per day
◯ 4–5 times per day
◯ 6 or more times per day

12. How often did you drink **liquor** or **mixed drinks**?

◯ NEVER

◯ 1 time per month or less
◯ 2–3 times per month
◯ 1–2 times per week
◯ 3–4 times per week
◯ 5–6 times per week
◯ 1 time per day
◯ 2–3 times per day
◯ 4–5 times per day
◯ 6 or more times per day

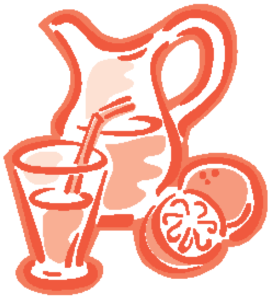

244004 - 2/6

Over the past 12 months...

13. Did you eat **oatmeal, grits,** or **other cooked cereal?**

- ◯ NO **(GO TO QUESTION 14)**
- ◯ YES

13a. How often did you eat **oatmeal, grits,** or **other cooked cereal IN THE WINTER**?

- ◯ NEVER
- ◯ 1–6 times per winter
- ◯ 7–11 times per winter
- ◯ 1 time per month
- ◯ 2–3 times per month
- ◯ 1 time per week
- ◯ 2 times per week
- ◯ 3–4 times per week
- ◯ 5–6 times per week
- ◯ 1 time per day
- ◯ 2 or more times per day

13b. How often did you eat **oatmeal, grits,** or **other cooked cereal DURING THE REST OF THE YEAR?**

- ◯ NEVER
- ◯ 1–6 times per year
- ◯ 7–11 times per year
- ◯ 1 time per month
- ◯ 2–3 times per month
- ◯ 1 time per week
- ◯ 2 times per week
- ◯ 3–4 times per week
- ◯ 5–6 times per week
- ◯ 1 time per day
- ◯ 2 or more times per day

13c. How often was the cooked cereal you ate **oatmeal**?

- ◯ Almost never or never
- ◯ About 1/4 of the time
- ◯ About 1/2 of the time
- ◯ About 3/4 of the time
- ◯ Almost always or always

14. How often did you eat **cold cereal**?

- ◯ NEVER **(GO TO QUESTION 15)**
- ◯ 1–6 times per year
- ◯ 7–11 times per year
- ◯ 1 time per month
- ◯ 2–3 times per month
- ◯ 1 time per week
- ◯ 2 times per week
- ◯ 3–4 times per week
- ◯ 5–6 times per week
- ◯ 1 time per day
- ◯ 2 or more times per day

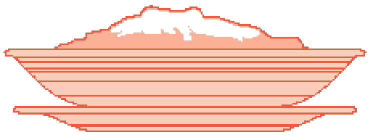

Question 15 appears in the next column.

14a. How often was the cold cereal you ate a **whole grain type** (such as shredded wheat, Wheaties, Cheerios, Raisin Bran or other bran, oat, or whole wheat cereal)?

- ◯ Almost never or never
- ◯ About 1/4 of the time
- ◯ About 1/2 of the time
- ◯ About 3/4 of the time
- ◯ Almost always or always

14b. Was **milk** added to your cold cereal?

- ◯ NO **(GO TO QUESTION 15)**
- ◯ YES

14c. What kind of **milk** was usually added?

- ◯ Whole milk
- ◯ 2% fat milk
- ◯ 1% fat milk
- ◯ Skim, nonfat, or 1/2% fat milk
- ◯ Soy milk
- ◯ Rice milk
- ◯ Raw, unpasteurized milk
- ◯ Other

15. How often did you eat **applesauce**?

- ◯ NEVER
- ◯ 1–6 times per year
- ◯ 7–11 times per year
- ◯ 1 time per month
- ◯ 2–3 times per month
- ◯ 1 time per week
- ◯ 2 times per week
- ◯ 3–4 times per week
- ◯ 5–6 times per week
- ◯ 1 time per day
- ◯ 2 or more times per day

16. How often did you eat **apples**?

- ◯ NEVER
- ◯ 1–6 times per year
- ◯ 7–11 times per year
- ◯ 1 time per month
- ◯ 2–3 times per month
- ◯ 1 time per week
- ◯ 2 times per week
- ◯ 3–4 times per week
- ◯ 5–6 times per week
- ◯ 1 time per day
- ◯ 2 or more times per day

17. How often did you eat **pears** (fresh, canned, or frozen)?

- ◯ NEVER
- ◯ 1–6 times per year
- ◯ 7–11 times per year
- ◯ 1 time per month
- ◯ 2–3 times per month
- ◯ 1 time per week
- ◯ 2 times per week
- ◯ 3–4 times per week
- ◯ 5–6 times per week
- ◯ 1 time per day
- ◯ 2 or more times per day

Over the past 12 months...

18. How often did you eat **bananas** ?

- ◯ NEVER
- ◯ 1–6 times per year
- ◯ 7–11 times per year
- ◯ 1 time per month
- ◯ 2–3 times per month
- ◯ 1 time per week
- ◯ 2 times per week
- ◯ 3–4 times per week
- ◯ 5–6 times per week
- ◯ 1 time per day
- ◯ 2 or more times per day

19. How often did you eat **pineapple**?

- ◯ NEVER
- ◯ 1–6 times per year
- ◯ 7–11 times per year
- ◯ 1 time per month
- ◯ 2–3 times per month
- ◯ 1 time per week
- ◯ 2 times per week
- ◯ 3–4 times per week
- ◯ 5–6 times per week
- ◯ 1 time per day
- ◯ 2 or more times per day

20. How often did you eat **dried fruit**, such as prunes or raisins?

- ◯ NEVER
- ◯ 1–6 times per year
- ◯ 7–11 times per year
- ◯ 1 time per month
- ◯ 2–3 times per month
- ◯ 1 time per week
- ◯ 2 times per week
- ◯ 3–4 times per week
- ◯ 5–6 times per week
- ◯ 1 time per day
- ◯ 2 or more times per day

21. Over the past 12 months, did you eat **peaches, nectarines,** or **plums**?

- ◯ NO **(GO TO QUESTION 22)**
- ◯ YES

21a. How often did you eat **fresh peaches**, **nectarines**, or **plums WHEN IN SEASON**?

- ◯ NEVER
- ◯ 1–6 times per season
- ◯ 7–11 times per season
- ◯ 1 time per month
- ◯ 2–3 times per month
- ◯ 1 time per week
- ◯ 2 times per week
- ◯ 3–4 times per week
- ◯ 5–6 times per week
- ◯ 1 time per day
- ◯ 2 or more times per day

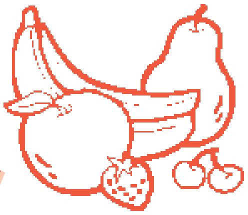

Question 22 appears in the next column.

21b. How often did you eat **peaches, nectarines,** or **plums** (fresh, canned, or frozen) **DURING THE REST OF THE YEAR**?

- ◯ NEVER
- ◯ 1–6 times per year
- ◯ 7–11 times per year
- ◯ 1 time per month
- ◯ 2–3 times per month
- ◯ 1 time per week
- ◯ 2 times per week
- ◯ 3–4 times per week
- ◯ 5–6 times per week
- ◯ 1 time per day
- ◯ 2 or more times per day

22. How often did you eat **grapes**?

- ◯ NEVER
- ◯ 1–6 times per year
- ◯ 7–11 times per year
- ◯ 1 time per month
- ◯ 2–3 times per month
- ◯ 1 time per week
- ◯ 2 times per week
- ◯ 3–4 times per week
- ◯ 5–6 times per week
- ◯ 1 time per day
- ◯ 2 or more times per day

23. Over the past 12 months, did you eat **melons** (such as cantaloupe, watermelon, or honeydew)?

- ◯ NO **(GO TO QUESTION 24)**
- ◯ YES

23a. How often did you eat **fresh melons** (such as cantaloupe, watermelon, or honeydew) **WHEN IN SEASON**?

- ◯ NEVER
- ◯ 1–6 times per season
- ◯ 7–11 times per season
- ◯ 1 time per month
- ◯ 2–3 times per month
- ◯ 1 time per week
- ◯ 2 times per week
- ◯ 3–4 times per week
- ◯ 5–6 times per week
- ◯ 1 time per day
- ◯ 2 or more times per day

23b. How often did you eat **fresh or frozen melons** (such as cantaloupe, watermelon, or honeydew) **DURING THE REST OF THE YEAR**?

- ◯ NEVER
- ◯ 1–6 times per year
- ◯ 7–11 times per year
- ◯ 1 time per month
- ◯ 2–3 times per month
- ◯ 1 time per week
- ◯ 2 times per week
- ◯ 3–4 times per week
- ◯ 5–6 times per week
- ◯ 1 time per day
- ◯ 2 or more times per day

Question 24 appears on the next page.

244004 - 3/6

Over the past 12 months...

24. Did you eat **strawberries**?

- ◯ NO **(GO TO QUESTION 25)**
- ◯ YES

24a. How often did you eat **fresh strawberries WHEN IN SEASON**?

- ◯ NEVER

◯ 1–6 times per season	◯ 2 times per week
◯ 7–11 times per season	◯ 3–4 times per week
◯ 1 time per month	◯ 5–6 times per week
◯ 2–3 times per month	◯ 1 time per day
◯ 1 time per week	◯ 2 or more times per day

24b. How often did you eat **fresh** or **frozen strawberries DURING THE REST OF THE YEAR**?

- ◯ NEVER

◯ 1–6 times per year	◯ 2 times per week
◯ 7–11 times per year	◯ 3–4 times per week
◯ 1 time per month	◯ 5–6 times per week
◯ 2–3 times per month	◯ 1 time per day
◯ 1 time per week	◯ 2 or more times per day

25. Over the past 12 months, did you eat **oranges, tangerines, clementines,** or **tangelos**?

- ◯ NO **(GO TO QUESTION 26)**
- ◯ YES

25a. How often did you eat **fresh oranges, tangerines, clementines,** or **tangelos WHEN IN SEASON**?

- ◯ NEVER

◯ 1–6 times per season	◯ 2 times per week
◯ 7–11 times per season	◯ 3–4 times per week
◯ 1 time per month	◯ 5–6 times per week
◯ 2–3 times per month	◯ 1 time per day
◯ 1 time per week	◯ 2 or more times per day

Question 26 appears in the next column.

25b. How often did you eat **oranges, tangerines, clementines,** or **tangelos** (fresh or canned) **DURING THE REST OF THE YEAR**?

- ◯ NEVER

◯ 1–6 times per year	◯ 2 times per week
◯ 7–11 times per year	◯ 3–4 times per week
◯ 1 time per month	◯ 5–6 times per week
◯ 2–3 times per month	◯ 1 time per day
◯ 1 time per week	◯ 2 or more times per day

26. Over the past 12 months, did you eat **grapefruit**?

- ◯ NO **(GO TO QUESTION 27)**
- ◯ YES

26a. How often did you eat **fresh grapefruit WHEN IN SEASON**?

- ◯ NEVER

◯ 1–6 times per season	◯ 2 times per week
◯ 7–11 times per season	◯ 3–4 times per week
◯ 1 time per month	◯ 5–6 times per week
◯ 2–3 times per month	◯ 1 time per day
◯ 1 time per week	◯ 2 or more times per day

26b. How often did you eat **grapefruit** (fresh or canned) **DURING THE REST OF THE YEAR?**

- ◯ NEVER

◯ 1–6 times per year	◯ 2 times per week
◯ 7–11 times per year	◯ 3–4 times per week
◯ 1 time per month	◯ 5–6 times per week
◯ 2–3 times per month	◯ 1 time per day
◯ 1 time per week	◯ 2 or more times per day

27. How often did you eat **other kinds of fruit**?

- ◯ NEVER

◯ 1–6 times per year	◯ 2 times per week
◯ 7–11 times per year	◯ 3–4 times per week
◯ 1 time per month	◯ 5–6 times per week
◯ 2–3 times per month	◯ 1 time per day
◯ 1 time per week	◯ 2 or more times per day

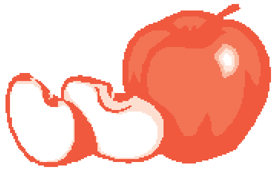

3/8" SPINE PERF

Over the <u>past 12 months</u>...

28. How often did you eat **COOKED greens** (such as spinach, turnip, collard, mustard, chard, or kale)?

- ◯ NEVER

◯ 1–6 times per year	◯ 2 times per week
◯ 7–11 times per year	◯ 3–4 times per week
◯ 1 time per month	◯ 5–6 times per week
◯ 2–3 times per month	◯ 1 time per day
◯ 1 time per week	◯ 2 or more times per day

29. How often did you eat **RAW greens** (such as spinach, turnip, collard, mustard, chard, or kale)? (*We will ask about lettuce later.*)

- ◯ NEVER

◯ 1–6 times per year	◯ 2 times per week
◯ 7–11 times per year	◯ 3–4 times per week
◯ 1 time per month	◯ 5–6 times per week
◯ 2–3 times per month	◯ 1 time per day
◯ 1 time per week	◯ 2 or more times per day

30. How often did you eat **coleslaw**?

- ◯ NEVER

◯ 1–6 times per year	◯ 2 times per week
◯ 7–11 times per year	◯ 3–4 times per week
◯ 1 time per month	◯ 5–6 times per week
◯ 2–3 times per month	◯ 1 time per day
◯ 1 time per week	◯ 2 or more times per day

31. How often did you eat **sauerkraut** or **cabbage** (other than coleslaw)?

- ◯ NEVER

◯ 1–6 times per year	◯ 2 times per week
◯ 7–11 times per year	◯ 3–4 times per week
◯ 1 time per month	◯ 5–6 times per week
◯ 2–3 times per month	◯ 1 time per day
◯ 1 time per week	◯ 2 or more times per day

32. How often did you eat **carrots** (fresh, canned, or frozen)?

- ◯ NEVER

◯ 1–6 times per year	◯ 2 times per week
◯ 7–11 times per year	◯ 3–4 times per week
◯ 1 time per month	◯ 5–6 times per week
◯ 2–3 times per month	◯ 1 time per day
◯ 1 time per week	◯ 2 or more times per day

33. How often did you eat **string beans** or **green beans** (fresh, canned, or frozen)?

- ◯ NEVER

◯ 1–6 times per year	◯ 2 times per week
◯ 7–11 times per year	◯ 3–4 times per week
◯ 1 time per month	◯ 5–6 times per week
◯ 2–3 times per month	◯ 1 time per day
◯ 1 time per week	◯ 2 or more times per day

34. How often did you eat **peas** (fresh, canned, or frozen)?

- ◯ NEVER

◯ 1–6 times per year	◯ 2 times per week
◯ 7–11 times per year	◯ 3–4 times per week
◯ 1 time per month	◯ 5–6 times per week
◯ 2–3 times per month	◯ 1 time per day
◯ 1 time per week	◯ 2 or more times per day

35. Over the <u>past 12 months</u>, did you eat **corn**?

- ◯ NO **(GO TO QUESTION 36)**
- ◯ YES

35a. How often did you eat **corn** (fresh, canned, or frozen) **WHEN IN SEASON**?

- ◯ NEVER

◯ 1–6 times per season	◯ 2 times per week
◯ 7–11 times per season	◯ 3–4 times per week
◯ 1 time per month	◯ 5–6 times per week
◯ 2–3 times per month	◯ 1 time per day
◯ 1 time per week	◯ 2 or more times per day

35b. How often did you eat **corn** (fresh, canned, or frozen) **DURING THE REST OF THE YEAR**?

- ◯ NEVER

◯ 1–6 times per year	◯ 2 times per week
◯ 7–11 times per year	◯ 3–4 times per week
◯ 1 time per month	◯ 5–6 times per week
◯ 2–3 times per month	◯ 1 time per day
◯ 1 time per week	◯ 2 or more times per day

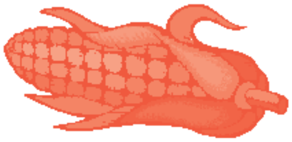

Question 36 appears on the next page.

244004 - 4/6

Over the past 12 months...

36. How often did you eat **broccoli** (fresh or frozen)?

◯ NEVER

◯ 1–6 times per year
◯ 7–11 times per year
◯ 1 time per month
◯ 2–3 times per month
◯ 1 time per week
◯ 2 times per week
◯ 3–4 times per week
◯ 5–6 times per week
◯ 1 time per day
◯ 2 or more times per day

37. How often did you eat **cauliflower** or **Brussels sprouts** (fresh or frozen)?

◯ NEVER

◯ 1–6 times per year
◯ 7–11 times per year
◯ 1 time per month
◯ 2–3 times per month
◯ 1 time per week
◯ 2 times per week
◯ 3–4 times per week
◯ 5–6 times per week
◯ 1 time per day
◯ 2 or more times per day

38. How often did you eat **mixed vegetables**?

◯ NEVER

◯ 1–6 times per year
◯ 7–11 times per year
◯ 1 time per month
◯ 2–3 times per month
◯ 1 time per week
◯ 2 times per week
◯ 3–4 times per week
◯ 5–6 times per week
◯ 1 time per day
◯ 2 or more times per day

39. How often did you eat **onions** (including in mixtures)?

◯ NEVER

◯ 1–6 times per year
◯ 7–11 times per year
◯ 1 time per month
◯ 2–3 times per month
◯ 1 time per week
◯ 2 times per week
◯ 3–4 times per week
◯ 5–6 times per week
◯ 1 time per day
◯ 2 or more times per day

40. Over the past 12 months, how often did you eat **sweet or hot peppers** (green, red, or yellow)?

◯ NEVER

◯ 1–6 times per year
◯ 7–11 times per year
◯ 1 time per month
◯ 2–3 times per month
◯ 1 time per week
◯ 2 times per week
◯ 3–4 times per week
◯ 5–6 times per week
◯ 1 time per day
◯ 2 or more times per day

41. How often did you eat **raw cucumbers** (not including pickles)?

◯ NEVER

◯ 1–6 times per year
◯ 7–11 times per year
◯ 1 time per month
◯ 2–3 times per month
◯ 1 time per week
◯ 2 times per week
◯ 3–4 times per week
◯ 5–6 times per week
◯ 1 time per day
◯ 2 or more times per day

42. Over the past 12 months, did you eat **fresh tomatoes** (including those in salads)?

◯ NO **(GO TO QUESTION 43)**

◯ YES

42a. How often did you eat **fresh tomatoes** (including those in salads) **WHEN IN SEASON**?

◯ NEVER

◯ 1–6 times per season
◯ 7–11 times per season
◯ 1 time per month
◯ 2–3 times per month
◯ 1 time per week
◯ 2 times per week
◯ 3–4 times per week
◯ 5–6 times per week
◯ 1 time per day
◯ 2 or more times per day

42b. How often did you eat **fresh tomatoes** (including those in salads) **DURING THE REST OF THE YEAR**?

◯ NEVER

◯ 1–6 times per year
◯ 7–11 times per year
◯ 1 time per month
◯ 2–3 times per month
◯ 1 time per week
◯ 2 times per week
◯ 3–4 times per week
◯ 5–6 times per week
◯ 1 time per day
◯ 2 or more times per day

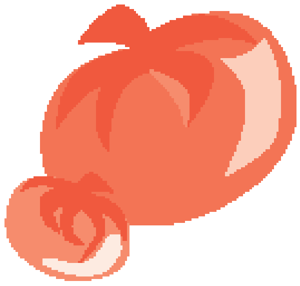

Question 43 appears on the next page.

3/8" SPINE PERF

Over the past 12 months...

43. Did you eat **summer squash** (include yellow and green squash)?

◯ NO **(GO TO QUESTION 44)**

◯ YES

43a. How often did you eat **summer squash WHEN IN SEASON** (include yellow and green squash)?

◯ NEVER

◯ 1–6 times per season
◯ 7–11 times per season
◯ 1 time per month
◯ 2–3 times per month
◯ 1 time per week
◯ 2 times per week
◯ 3–4 times per week
◯ 5–6 times per week
◯ 1 time per day
◯ 2 or more times per day

43b. How often did you eat **summer squash DURING THE REST OF THE YEAR** (include yellow and green squash)?

◯ NEVER

◯ 1–6 times per year
◯ 7–11 times per year
◯ 1 time per month
◯ 2–3 times per month
◯ 1 time per week
◯ 2 times per week
◯ 3–4 times per week
◯ 5–6 times per week
◯ 1 time per day
◯ 2 or more times per day

44. How often did you eat **lettuce salads** (with or without other vegetables)?

◯ NEVER **(GO TO QUESTION 45)**

◯ 1–6 times per year
◯ 7–11 times per year
◯ 1 time per month
◯ 2–3 times per month
◯ 1 time per week
◯ 2 times per week
◯ 3–4 times per week
◯ 5–6 times per week
◯ 1 time per day
◯ 2 or more times per day

44a. How often were the lettuce salads you ate made with **dark green leaves**?

◯ Almost never or never
◯ About 1/4 of the time
◯ About 1/2 of the time
◯ About 3/4 of the time
◯ Almost always or always

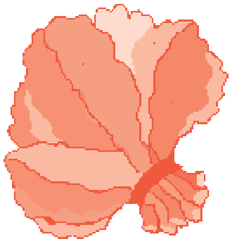

Question 45 appears in the next column.

45. How often did you eat **salad dressing** (including low-fat) on salads or other vegetables?

◯ NEVER

◯ 1–6 times per year
◯ 7–11 times per year
◯ 1 time per month
◯ 2–3 times per month
◯ 1 time per week
◯ 2 times per week
◯ 3–4 times per week
◯ 5–6 times per week
◯ 1 time per day
◯ 2 or more times per day

46. How often did you eat **sweet potatoes** or **yams**?

◯ NEVER

◯ 1–6 times per year
◯ 7–11 times per year
◯ 1 time per month
◯ 2–3 times per month
◯ 1 time per week
◯ 2 times per week
◯ 3–4 times per week
◯ 5–6 times per week
◯ 1 time per day
◯ 2 or more times per day

47. How often did you eat **French fries, home fries, hash browned potatoes**, or **tater tots**?

◯ NEVER

◯ 1–6 times per year
◯ 7–11 times per year
◯ 1 time per month
◯ 2–3 times per month
◯ 1 time per week
◯ 2 times per week
◯ 3–4 times per week
◯ 5–6 times per week
◯ 1 time per day
◯ 2 or more times per day

48. How often did you eat **potato salad**?

◯ NEVER

◯ 1–6 times per year
◯ 7–11 times per year
◯ 1 time per month
◯ 2–3 times per month
◯ 1 time per week
◯ 2 times per week
◯ 3–4 times per week
◯ 5–6 times per week
◯ 1 time per day
◯ 2 or more times per day

49. How often did you eat **baked, boiled,** or **mashed potatoes**?

◯ NEVER

◯ 1–6 times per year
◯ 7–11 times per year
◯ 1 time per month
◯ 2–3 times per month
◯ 1 time per week
◯ 2 times per week
◯ 3–4 times per week
◯ 5–6 times per week
◯ 1 time per day
◯ 2 or more times per day

244004 - 5/6

Over the past 12 months...

50. How often did you eat **salsa**?

- ◯ NEVER
- ◯ 1–6 times per year
- ◯ 7–11 times per year
- ◯ 1 time per month
- ◯ 2–3 times per month
- ◯ 1 time per week
- ◯ 2 times per week
- ◯ 3–4 times per week
- ◯ 5–6 times per week
- ◯ 1 time per day
- ◯ 2 or more times per day

51. How often did you eat **catsup**?

- ◯ NEVER
- ◯ 1–6 times per year
- ◯ 7–11 times per year
- ◯ 1 time per month
- ◯ 2–3 times per month
- ◯ 1 time per week
- ◯ 2 times per week
- ◯ 3–4 times per week
- ◯ 5–6 times per week
- ◯ 1 time per day
- ◯ 2 or more times per day

52. How often did you eat **pickles** or **pickled vegetables**?

- ◯ NEVER
- ◯ 1–6 times per year
- ◯ 7–11 times per year
- ◯ 1 time per month
- ◯ 2–3 times per month
- ◯ 1 time per week
- ◯ 2 times per week
- ◯ 3–4 times per week
- ◯ 5–6 times per week
- ◯ 1 time per day
- ◯ 2 or more times per day

53. How often did you eat **stuffing, dressing,** or **dumplings**?

- ◯ NEVER
- ◯ 1–6 times per year
- ◯ 7–11 times per year
- ◯ 1 time per month
- ◯ 2–3 times per month
- ◯ 1 time per week
- ◯ 2 times per week
- ◯ 3–4 times per week
- ◯ 5–6 times per week
- ◯ 1 time per day
- ◯ 2 or more times per day

54. How often did you eat **chili**?

- ◯ NEVER
- ◯ 1–6 times per year
- ◯ 7–11 times per year
- ◯ 1 time per month
- ◯ 2–3 times per month
- ◯ 1 time per week
- ◯ 2 times per week
- ◯ 3–4 times per week
- ◯ 5–6 times per week
- ◯ 1 time per day
- ◯ 2 or more times per day

55. How often did you eat **tortillas** or **tacos**?

- ◯ NEVER **(GO TO QUESTION 56)**
- ◯ 1–6 times per year
- ◯ 7–11 times per year
- ◯ 1 time per month
- ◯ 2–3 times per month
- ◯ 1 time per week
- ◯ 2 times per week
- ◯ 3–4 times per week
- ◯ 5–6 times per week
- ◯ 1 time per day
- ◯ 2 or more times per day

55a. How often were your tortillas or tacos **corn tortillas** or **tacos**?

- ◯ Almost never or never
- ◯ About 1/4 of the time
- ◯ About 1/2 of the time
- ◯ About 3/4 of the time
- ◯ Almost always or always

56. How often did you eat **cooked dried beans** (such as baked beans, pintos, kidney, blackeyed peas, lima, lentils, soybeans, or refried beans)? *(Please don't include bean soups or chili.)*

- ◯ NEVER
- ◯ 1–6 times per year
- ◯ 7–11 times per year
- ◯ 1 time per month
- ◯ 2–3 times per month
- ◯ 1 time per week
- ◯ 2 times per week
- ◯ 3–4 times per week
- ◯ 5–6 times per week
- ◯ 1 time per day
- ◯ 2 or more times per day

57. How often did you eat **other kinds of vegetables**?

- ◯ NEVER
- ◯ 1–6 times per year
- ◯ 7–11 times per year
- ◯ 1 time per month
- ◯ 2–3 times per month
- ◯ 1 time per week
- ◯ 2 times per week
- ◯ 3–4 times per week
- ◯ 5–6 times per week
- ◯ 1 time per day
- ◯ 2 or more times per day

58. How often did you eat **rice** or **other cooked grains** (such as bulgur, cracked wheat, or millet)?

- ◯ NEVER **(GO TO QUESTION 59)**
- ◯ 1–6 times per year
- ◯ 7–11 times per year
- ◯ 1 time per month
- ◯ 2–3 times per month
- ◯ 1 time per week
- ◯ 2 times per week
- ◯ 3–4 times per week
- ◯ 5–6 times per week
- ◯ 1 time per day
- ◯ 2 or more times per day

Question 59 appears on the next page.

Over the past 12 months...

58a. How often was the rice or other cooked grains you ate **brown rice, cracked wheat,** or **millet**?

- ◯ Almost never or never
- ◯ About 1/4 of the time
- ◯ About 1/2 of the time
- ◯ About 3/4 of the time
- ◯ Almost always or always

59. How often did you eat **pancakes, waffles,** or **French toast**?

◯ NEVER **(GO TO QUESTION 60)**

◯ 1–6 times per year	◯ 2 times per week
◯ 7–11 times per year	◯ 3–4 times per week
◯ 1 time per month	◯ 5–6 times per week
◯ 2–3 times per month	◯ 1 time per day
◯ 1 time per week	◯ 2 or more times per day

59a. How often was **syrup** added to your pancakes, waffles, or French toast?

- ◯ Almost never or never
- ◯ About 1/4 of the time
- ◯ About 1/2 of the time
- ◯ About 3/4 of the time
- ◯ Almost always or always

60. How often did you eat **lasagna, stuffed shells, stuffed manicotti, ravioli,** or **tortellini**? *(Please do not include spaghetti or other pasta.)*

◯ NEVER

◯ 1–6 times per year	◯ 2 times per week
◯ 7–11 times per year	◯ 3–4 times per week
◯ 1 time per month	◯ 5–6 times per week
◯ 2–3 times per month	◯ 1 time per day
◯ 1 time per week	◯ 2 or more times per day

61. How often did you eat **macaroni and cheese**?

◯ NEVER

◯ 1–6 times per year	◯ 2 times per week
◯ 7–11 times per year	◯ 3–4 times per week
◯ 1 time per month	◯ 5–6 times per week
◯ 2–3 times per month	◯ 1 time per day
◯ 1 time per week	◯ 2 or more times per day

62. How often did you eat **pasta salad** or **macaroni salad**?

◯ NEVER

◯ 1–6 times per year	◯ 2 times per week
◯ 7–11 times per year	◯ 3–4 times per week
◯ 1 time per month	◯ 5–6 times per week
◯ 2–3 times per month	◯ 1 time per day
◯ 1 time per week	◯ 2 or more times per day

63. Other than the pastas listed in Questions 60, 61, and 62, how often did you eat **pasta, spaghetti,** or **other noodles**?

◯ NEVER **(GO TO QUESTION 64)**

◯ 1–6 times per year	◯ 2 times per week
◯ 7–11 times per year	◯ 3–4 times per week
◯ 1 time per month	◯ 5–6 times per week
◯ 2–3 times per month	◯ 1 time per day
◯ 1 time per week	◯ 2 or more times per day

63a. How often did you eat your pasta, spaghetti, or other noodles with **tomato sauce** or **spaghetti sauce made WITH meat**?

- ◯ Almost never or never
- ◯ About 1/4 of the time
- ◯ About 1/2 of the time
- ◯ About 3/4 of the time
- ◯ Almost always or always

63b. How often did you eat your pasta, spaghetti, or other noodles with **tomato sauce** or **spaghetti sauce made WITHOUT meat**?

- ◯ Almost never or never
- ◯ About 1/4 of the time
- ◯ About 1/2 of the time
- ◯ About 3/4 of the time
- ◯ Almost always or always

63c. How often did you eat your pasta, spaghetti, or other noodles with **margarine, butter, oil,** or **cream sauce**?

- ◯ Almost never or never
- ◯ About 1/4 of the time
- ◯ About 1/2 of the time
- ◯ About 3/4 of the time
- ◯ Almost always or always

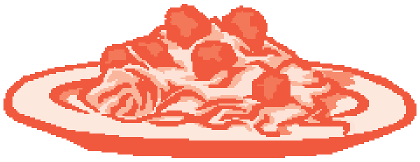

Question 64 appears on the next page.

244004 - 6/6

Over the <u>past 12 months</u>...

64. How often did you eat **bagels** or **English muffins**?

- ◯ NEVER
- ◯ 1–6 times per year
- ◯ 7–11 times per year
- ◯ 1 time per month
- ◯ 2–3 times per month
- ◯ 1 time per week
- ◯ 2 times per week
- ◯ 3–4 times per week
- ◯ 5–6 times per week
- ◯ 1 time per day
- ◯ 2 or more times per day

The next questions ask about your intake of breads other than bagels or English muffins. First, we will ask about bread you ate as part of sandwiches only. Then we will ask about all other bread you ate.

65. How often did you eat **breads** or **rolls AS PART OF SANDWICHES** (including burger and hot dog rolls)?

- ◯ NEVER **(GO TO QUESTION 66)**
- ◯ 1–6 times per year
- ◯ 7–11 times per year
- ◯ 1 time per month
- ◯ 2–3 times per month
- ◯ 1 time per week
- ◯ 2 times per week
- ◯ 3–4 times per week
- ◯ 5–6 times per week
- ◯ 1 time per day
- ◯ 2 or more times per day

65a. How often were the breads or rolls that you used for your sandwiches **white bread** (including burger and hot dog rolls)?

- ◯ Almost never or never
- ◯ About 1/4 of the time
- ◯ About 1/2 of the time
- ◯ About 3/4 of the time
- ◯ Almost always or always

66. How often did you eat **breads** or **dinner rolls, NOT AS PART OF SANDWICHES**?

- ◯ NEVER **(GO TO QUESTION 67)**
- ◯ 1–6 times per year
- ◯ 7–11 times per year
- ◯ 1 time per month
- ◯ 2–3 times per month
- ◯ 1 time per week
- ◯ 2 times per week
- ◯ 3–4 times per week
- ◯ 5–6 times per week
- ◯ 1 time per day
- ◯ 2 or more times per day

66a. How often were the breads or rolls you ate **white bread**?

- ◯ Almost never or never
- ◯ About 1/4 of the time
- ◯ About 1/2 of the time
- ◯ About 3/4 of the time
- ◯ Almost always or always

Question 67 appears in the next column.

67. How often did you eat **jam, jelly,** or **honey** on bagels, muffins, bread, rolls, or crackers?

- ◯ NEVER
- ◯ 1–6 times per year
- ◯ 7–11 times per year
- ◯ 1 time per month
- ◯ 2–3 times per month
- ◯ 1 time per week
- ◯ 2 times per week
- ◯ 3–4 times per week
- ◯ 5–6 times per week
- ◯ 1 time per day
- ◯ 2 or more times per day

68. How often did you eat **peanut butter** or **other nut butter**?

- ◯ NEVER
- ◯ 1–6 times per year
- ◯ 7–11 times per year
- ◯ 1 time per month
- ◯ 2–3 times per month
- ◯ 1 time per week
- ◯ 2 times per week
- ◯ 3–4 times per week
- ◯ 5–6 times per week
- ◯ 1 time per day
- ◯ 2 or more times per day

69. How often did you eat **roast beef** or **steak IN SANDWICHES**?

- ◯ NEVER
- ◯ 1–6 times per year
- ◯ 7–11 times per year
- ◯ 1 time per month
- ◯ 2–3 times per month
- ◯ 1 time per week
- ◯ 2 times per week
- ◯ 3–4 times per week
- ◯ 5–6 times per week
- ◯ 1 time per day
- ◯ 2 or more times per day

70. How often did you eat **turkey** or **chicken COLD CUTS** (such as loaf, luncheon meat, turkey ham, turkey salami, or turkey pastrami)? (*We will ask about other turkey or chicken later.)*

- ◯ NEVER
- ◯ 1–6 times per year
- ◯ 7–11 times per year
- ◯ 1 time per month
- ◯ 2–3 times per month
- ◯ 1 time per week
- ◯ 2 times per week
- ◯ 3–4 times per week
- ◯ 5–6 times per week
- ◯ 1 time per day
- ◯ 2 or more times per day

71. How often did you eat **luncheon** or **deli-style ham**? *(We will ask about other ham later.)*

- ◯ NEVER **(GO TO QUESTION 72)**
- ◯ 1–6 times per year
- ◯ 7–11 times per year
- ◯ 1 time per month
- ◯ 2–3 times per month
- ◯ 1 time per week
- ◯ 2 times per week
- ◯ 3–4 times per week
- ◯ 5–6 times per week
- ◯ 1 time per day
- ◯ 2 or more times per day

Question 72 appears on the next page.

Over the past 12 months...

71a. How often was the luncheon or deli-style ham you ate **light, low-fat, or fat-free**?

- ◯ Almost never or never
- ◯ About 1/4 of the time
- ◯ About 1/2 of the time
- ◯ About 3/4 of the time
- ◯ Almost always or always

72. How often did you eat **other cold cuts** or **luncheon meats** (such as bologna, salami, corned beef, pastrami, or others, including low-fat)? *(Please do not include ham, turkey, or chicken cold cuts.)*

◯ NEVER **(GO TO QUESTION 73)**

◯ 1–6 times per year	◯ 2 times per week
◯ 7–11 times per year	◯ 3–4 times per week
◯ 1 time per month	◯ 5–6 times per week
◯ 2–3 times per month	◯ 1 time per day
◯ 1 time per week	◯ 2 or more times per day

72a. How often were the other cold cuts or luncheon meats you ate **light, low-fat,** or **fat-free**? *(Please do not include ham, turkey, or chicken cold cuts.)*

- ◯ Almost never or never
- ◯ About 1/4 of the time
- ◯ About 1/2 of the time
- ◯ About 3/4 of the time
- ◯ Almost always or always

73. How often did you eat **canned tuna** (including in salads, sandwiches, or casseroles)?

◯ NEVER

◯ 1–6 times per year	◯ 2 times per week
◯ 7–11 times per year	◯ 3–4 times per week
◯ 1 time per month	◯ 5–6 times per week
◯ 2–3 times per month	◯ 1 time per day
◯ 1 time per week	◯ 2 or more times per day

74. How often did you eat **GROUND chicken** or **turkey**? *(We will ask about other chicken and turkey later.)*

◯ NEVER

◯ 1–6 times per year	◯ 2 times per week
◯ 7–11 times per year	◯ 3–4 times per week
◯ 1 time per month	◯ 5–6 times per week
◯ 2–3 times per month	◯ 1 time per day
◯ 1 time per week	◯ 2 or more times per day

75. How often did you eat **beef hamburgers** or **cheeseburgers**?

◯ NEVER **(GO TO QUESTION 76)**

◯ 1–6 times per year	◯ 2 times per week
◯ 7–11 times per year	◯ 3–4 times per week
◯ 1 time per month	◯ 5–6 times per week
◯ 2–3 times per month	◯ 1 time per day
◯ 1 time per week	◯ 2 or more times per day

75a. How often were the beef hamburgers or cheeseburgers you ate made with **lean ground beef**?

- ◯ Almost never or never
- ◯ About 1/4 of the time
- ◯ About 1/2 of the time
- ◯ About 3/4 of the time
- ◯ Almost always or always

76. How often did you eat **ground beef in mixtures** (such as meatballs, casseroles, chili, or meatloaf)?

◯ NEVER

◯ 1–6 times per year	◯ 2 times per week
◯ 7–11 times per year	◯ 3–4 times per week
◯ 1 time per month	◯ 5–6 times per week
◯ 2–3 times per month	◯ 1 time per day
◯ 1 time per week	◯ 2 or more times per day

77. How often did you eat **hot dogs** or **frankfurters**? *(Please do not include sausages or vegetarian hot dogs.)*

◯ NEVER **(GO TO QUESTION 78)**

◯ 1–6 times per year	◯ 2 times per week
◯ 7–11 times per year	◯ 3–4 times per week
◯ 1 time per month	◯ 5–6 times per week
◯ 2–3 times per month	◯ 1 time per day
◯ 1 time per week	◯ 2 or more times per day

77a. How often were the hot dogs or frankfurters you ate **light** or **low-fat hot dogs**?

- ◯ Almost never or never
- ◯ About 1/4 of the time
- ◯ About 1/2 of the time
- ◯ About 3/4 of the time
- ◯ Almost always or always

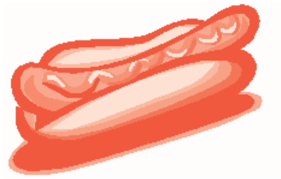

Question 78 appears on the next page.

3/8" SPINE PERF

Over the <u>past 12 months</u>...

78. How often did you eat beef mixtures such as **beef stew, beef pot pie, beef and noodles,** or **beef and vegetables**?

◯ NEVER

◯ 1–6 times per year
◯ 7–11 times per year
◯ 1 time per month
◯ 2–3 times per month
◯ 1 time per week
◯ 2 times per week
◯ 3–4 times per week
◯ 5–6 times per week
◯ 1 time per day
◯ 2 or more times per day

79. How often did you eat **roast beef** or **pot roast**? *(Please do not include roast beef or pot roast in sandwiches.)*

◯ NEVER

◯ 1–6 times per year
◯ 7–11 times per year
◯ 1 time per month
◯ 2–3 times per month
◯ 1 time per week
◯ 2 times per week
◯ 3–4 times per week
◯ 5–6 times per week
◯ 1 time per day
◯ 2 or more times per day

80. How often did you eat **steak** (beef)? (*Do not include steak in sandwiches*)

◯ NEVER **(GO TO QUESTION 81)**

◯ 1–6 times per year
◯ 7–11 times per year
◯ 1 time per month
◯ 2–3 times per month
◯ 1 time per week
◯ 2 times per week
◯ 3–4 times per week
◯ 5–6 times per week
◯ 1 time per day
◯ 2 or more times per day

80a. How often was the steak you ate **lean steak**?

◯ Almost never or never
◯ About 1/4 of the time
◯ About 1/2 of the time
◯ About 3/4 of the time
◯ Almost always or always

81. How often did you eat **pork** or **beef spareribs**?

◯ NEVER

◯ 1–6 times per year
◯ 7–11 times per year
◯ 1 time per month
◯ 2–3 times per month
◯ 1 time per week
◯ 2 times per week
◯ 3–4 times per week
◯ 5–6 times per week
◯ 1 time per day
◯ 2 or more times per day

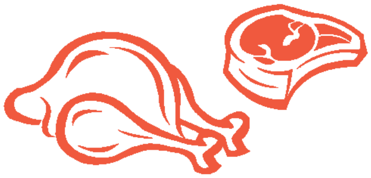

82. How often did you eat **roast turkey, turkey cutlets,** or **turkey nuggets** (including in sandwiches)?

◯ NEVER

◯ 1–6 times per year
◯ 7–11 times per year
◯ 1 time per month
◯ 2–3 times per month
◯ 1 time per week
◯ 2 times per week
◯ 3–4 times per week
◯ 5–6 times per week
◯ 1 time per day
◯ 2 or more times per day

83. How often did you eat **chicken** as part of **salads, sandwiches, casseroles, stews,** or **other mixtures**?

◯ NEVER

◯ 1–6 times per year
◯ 7–11 times per year
◯ 1 time per month
◯ 2–3 times per month
◯ 1 time per week
◯ 2 times per week
◯ 3–4 times per week
◯ 5–6 times per week
◯ 1 time per day
◯ 2 or more times per day

84. How often did you eat **baked, broiled, roasted, stewed,** or **fried chicken** (including nuggets)? *(Please do not include chicken in mixtures.)*

◯ NEVER **(GO TO QUESTION 85)**

◯ 1–6 times per year
◯ 7–11 times per year
◯ 1 time per month
◯ 2–3 times per month
◯ 1 time per week
◯ 2 times per week
◯ 3–4 times per week
◯ 5–6 times per week
◯ 1 time per day
◯ 2 or more times per day

84a. How often was the chicken you ate **fried chicken** (including deep fried) or **chicken nuggets**?

◯ Almost never or never
◯ About 1/4 of the time
◯ About 1/2 of the time
◯ About 3/4 of the time
◯ Almost always or always

84b. How often was the chicken you ate **WHITE meat**?

◯ Almost never or never
◯ About 1/4 of the time
◯ About 1/2 of the time
◯ About 3/4 of the time
◯ Almost always or always

Question 85 appears on the next page.

Over the <u>past 12 months</u>...

84c. How often did you eat chicken **WITH skin**?

- ◯ Almost never or never
- ◯ About 1/4 of the time
- ◯ About 1/2 of the time
- ◯ About 3/4 of the time
- ◯ Almost always or always

85. How often did you eat **baked ham** or **ham steak**?

◯ NEVER

◯ 1–6 times per year	◯ 2 times per week
◯ 7–11 times per year	◯ 3–4 times per week
◯ 1 time per month	◯ 5–6 times per week
◯ 2–3 times per month	◯ 1 time per day
◯ 1 time per week	◯ 2 or more times per day

86. How often did you eat **pork** (including chops, roasts, and in mixed dishes)? *(Please do not include ham, ham steak, or sausage.)*

◯ NEVER

◯ 1–6 times per year	◯ 2 times per week
◯ 7–11 times per year	◯ 3–4 times per week
◯ 1 time per month	◯ 5–6 times per week
◯ 2–3 times per month	◯ 1 time per day
◯ 1 time per week	◯ 2 or more times per day

87. How often did you eat **gravy** on meat, chicken, potatoes, rice, etc.?

◯ NEVER

◯ 1–6 times per year	◯ 2 times per week
◯ 7–11 times per year	◯ 3–4 times per week
◯ 1 time per month	◯ 5–6 times per week
◯ 2–3 times per month	◯ 1 time per day
◯ 1 time per week	◯ 2 or more times per day

88. How often did you eat **liver** (all kinds) or **liverwurst**?

◯ NEVER

◯ 1–6 times per year	◯ 2 times per week
◯ 7–11 times per year	◯ 3–4 times per week
◯ 1 time per month	◯ 5–6 times per week
◯ 2–3 times per month	◯ 1 time per day
◯ 1 time per week	◯ 2 or more times per day

89. How often did you eat **bacon** (including low-fat)?

◯ NEVER **(GO TO QUESTION 90)**

◯ 1–6 times per year	◯ 2 times per week
◯ 7–11 times per year	◯ 3–4 times per week
◯ 1 time per month	◯ 5–6 times per week
◯ 2–3 times per month	◯ 1 time per day
◯ 1 time per week	◯ 2 or more times per day

89a. How often was the bacon you ate **light, low-fat,** or **lean bacon**?

- ◯ Almost never or never
- ◯ About 1/4 of the time
- ◯ About 1/2 of the time
- ◯ About 3/4 of the time
- ◯ Almost always or always

90. How often did you eat **sausage** (including low-fat)?

◯ NEVER **(GO TO QUESTION 91)**

◯ 1–6 times per year	◯ 2 times per week
◯ 7–11 times per year	◯ 3–4 times per week
◯ 1 time per month	◯ 5–6 times per week
◯ 2–3 times per month	◯ 1 time per day
◯ 1 time per week	◯ 2 or more times per day

90a. How often was the sausage you ate **light, low-fat,** or **lean sausage**?

- ◯ Almost never or never
- ◯ About 1/4 of the time
- ◯ About 1/2 of the time
- ◯ About 3/4 of the time
- ◯ Almost always or always

91. How often did you eat **smoked fish** or **seafood** (such as smoked salmon, lox, or others)?

◯ NEVER

◯ 1–6 times per year	◯ 2 times per week
◯ 7–11 times per year	◯ 3–4 times per week
◯ 1 time per month	◯ 5–6 times per week
◯ 2–3 times per month	◯ 1 time per day
◯ 1 time per week	◯ 2 or more times per day

92. How often did you eat **sushi**?

◯ NEVER **(GO TO QUESTION 93)**

◯ 1–6 times per year	◯ 2 times per week
◯ 7–11 times per year	◯ 3–4 times per week
◯ 1 time per month	◯ 5–6 times per week
◯ 2–3 times per month	◯ 1 time per day
◯ 1 time per week	◯ 2 or more times per day

Question 93 appears on the next page.

3/8" SPINE PERF

Over the past 12 months...

92a. How often did the **sushi** you ate contain **raw fish** or **seafood** (including shellfish)?

- ◯ Almost never or never
- ◯ About 1/4 of the time
- ◯ About 1/2 of the time
- ◯ About 3/4 of the time
- ◯ Almost always or always

93. How often did you eat **raw oysters, raw clams,** or **other raw fish** (not including raw fish in sushi)?

◯ NEVER

◯ 1–6 times per year	◯ 2 times per week
◯ 7–11 times per year	◯ 3–4 times per week
◯ 1 time per month	◯ 5–6 times per week
◯ 2–3 times per month	◯ 1 time per day
◯ 1 time per week	◯ 2 or more times per day

94. How often did you eat **fish sticks** or **fried fish** (including fried seafood or shellfish)?

◯ NEVER

◯ 1–6 times per year	◯ 2 times per week
◯ 7–11 times per year	◯ 3–4 times per week
◯ 1 time per month	◯ 5–6 times per week
◯ 2–3 times per month	◯ 1 time per day
◯ 1 time per week	◯ 2 or more times per day

95. How often did you eat **all other fish** or **seafood** (including shellfish) that was **NOT FRIED, SMOKED,** or **RAW** ?

◯ NEVER

◯ 1–6 times per year	◯ 2 times per week
◯ 7–11 times per year	◯ 3–4 times per week
◯ 1 time per month	◯ 5–6 times per week
◯ 2–3 times per month	◯ 1 time per day
◯ 1 time per week	◯ 2 or more times per day

96. How often did you eat **tofu**, **soy burgers**, or **soy meat-substitutes**?

◯ NEVER

◯ 1–6 times per year	◯ 2 times per week
◯ 7–11 times per year	◯ 3–4 times per week
◯ 1 time per month	◯ 5–6 times per week
◯ 2–3 times per month	◯ 1 time per day
◯ 1 time per week	◯ 2 or more times per day

97. Over the past 12 months, did you eat **soups**?

◯ NO **(GO TO QUESTION 98)**

◯ YES

97a. How often did you eat **soup DURING THE WINTER**?

◯ NEVER

◯ 1–6 times per winter	◯ 2 times per week
◯ 7–11 times per winter	◯ 3–4 times per week
◯ 1 time per month	◯ 5–6 times per week
◯ 2–3 times per month	◯ 1 time per day
◯ 1 time per week	◯ 2 or more times per day

97b. How often did you eat **soup DURING THE REST OF THE YEAR**?

◯ NEVER

◯ 1–6 times per year	◯ 2 times per week
◯ 7–11 times per year	◯ 3–4 times per week
◯ 1 time per month	◯ 5–6 times per week
◯ 2–3 times per month	◯ 1 time per day
◯ 1 time per week	◯ 2 or more times per day

97c. How often were the soups you ate **bean soups**?

- ◯ Almost never or never
- ◯ About 1/4 of the time
- ◯ About 1/2 of the time
- ◯ About 3/4 of the time
- ◯ Almost always or always

97d. How often were the soups you ate **cream soups** (including chowders)?

- ◯ Almost never or never
- ◯ About 1/4 of the time
- ◯ About 1/2 of the time
- ◯ About 3/4 of the time
- ◯ Almost always or always

97e. How often were the soups you ate **tomato** or **vegetable soups**?

- ◯ Almost never or never
- ◯ About 1/4 of the time
- ◯ About 1/2 of the time
- ◯ About 3/4 of the time
- ◯ Almost always or always

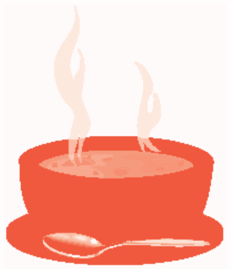

Question 98 appears on the next page.

Over the past 12 months...

97f. How often were the soups you ate **broth soups** (including chicken) **with** or **without noodles** or **rice**?

- ◯ Almost never or never
- ◯ About 1/4 of the time
- ◯ About 1/2 of the time
- ◯ About 3/4 of the time
- ◯ Almost always or always

98. How often did you eat **pizza**?

◯ NEVER **(GO TO QUESTION 99)**

◯ 1–6 times per year	◯ 2 times per week
◯ 7–11 times per year	◯ 3–4 times per week
◯ 1 time per month	◯ 5–6 times per week
◯ 2–3 times per month	◯ 1 time per day
◯ 1 time per week	◯ 2 or more times per day

98a. How often did you eat pizza with **pepperoni, sausage,** or **other meat**?

- ◯ Almost never or never
- ◯ About 1/4 of the time
- ◯ About 1/2 of the time
- ◯ About 3/4 of the time
- ◯ Almost always or always

99. How often did you eat **crackers**?

◯ NEVER

◯ 1–6 times per year	◯ 2 times per week
◯ 7–11 times per year	◯ 3–4 times per week
◯ 1 time per month	◯ 5–6 times per week
◯ 2–3 times per month	◯ 1 time per day
◯ 1 time per week	◯ 2 or more times per day

100. How often did you eat **corn bread** or **corn muffins**?

◯ NEVER

◯ 1–6 times per year	◯ 2 times per week
◯ 7–11 times per year	◯ 3–4 times per week
◯ 1 time per month	◯ 5–6 times per week
◯ 2–3 times per month	◯ 1 time per day
◯ 1 time per week	◯ 2 or more times per day

101. How often did you eat **biscuits**?

◯ NEVER

◯ 1–6 times per year	◯ 2 times per week
◯ 7–11 times per year	◯ 3–4 times per week
◯ 1 time per month	◯ 5–6 times per week
◯ 2–3 times per month	◯ 1 time per day
◯ 1 time per week	◯ 2 or more times per day

102. How often did you eat **potato chips** (including low-fat, fat-free, or low-salt)?

◯ NEVER **(GO TO QUESTION 103)**

◯ 1–6 times per year	◯ 2 times per week
◯ 7–11 times per year	◯ 3–4 times per week
◯ 1 time per month	◯ 5–6 times per week
◯ 2–3 times per month	◯ 1 time per day
◯ 1 time per week	◯ 2 or more times per day

102a. How often were the potato chips you ate **low-fat** or **fat-free chips**?

- ◯ Almost never or never
- ◯ About 1/4 of the time
- ◯ About 1/2 of the time
- ◯ About 3/4 of the time
- ◯ Almost always or always

103. How often did you eat **tortilla chips** or **corn chips** (including low-fat, fat-free, or low-salt)?

◯ NEVER **(GO TO QUESTION 104)**

◯ 1–6 times per year	◯ 2 times per week
◯ 7–11 times per year	◯ 3–4 times per week
◯ 1 time per month	◯ 5–6 times per week
◯ 2–3 times per month	◯ 1 time per day
◯ 1 time per week	◯ 2 or more times per day

103a. How often were the tortilla or corn chips you ate **low-fat** or **fat-free chips**?

- ◯ Almost never or never
- ◯ About 1/4 of the time
- ◯ About 1/2 of the time
- ◯ About 3/4 of the time
- ◯ Almost always or always

104. How often did you eat **popcorn** (including low-fat)?

◯ NEVER

◯ 1–6 times per year	◯ 2 times per week
◯ 7–11 times per year	◯ 3–4 times per week
◯ 1 time per month	◯ 5–6 times per week
◯ 2–3 times per month	◯ 1 time per day
◯ 1 time per week	◯ 2 or more times per day

105. How often did you eat **pretzels**?

◯ NEVER

◯ 1–6 times per year	◯ 2 times per week
◯ 7–11 times per year	◯ 3–4 times per week
◯ 1 time per month	◯ 5–6 times per week
◯ 2–3 times per month	◯ 1 time per day
◯ 1 time per week	◯ 2 or more times per day

3/8" SPINE PERF

Over the <u>past 12 months</u>...

106. How often did you eat **peanuts, walnuts, seeds,** or **other nuts**?

- ◯ NEVER

- ◯ 1–6 times per year
- ◯ 7–11 times per year
- ◯ 1 time per month
- ◯ 2–3 times per month
- ◯ 1 time per week
- ◯ 2 times per week
- ◯ 3–4 times per week
- ◯ 5–6 times per week
- ◯ 1 time per day
- ◯ 2 or more times per day

107. How often did you eat **granola bars**?

- ◯ NEVER

- ◯ 1–6 times per year
- ◯ 7–11 times per year
- ◯ 1 time per month
- ◯ 2–3 times per month
- ◯ 1 time per week
- ◯ 2 times per week
- ◯ 3–4 times per week
- ◯ 5–6 times per week
- ◯ 1 time per day
- ◯ 2 or more times per day

108. How often did you eat **yogurt** (NOT including frozen yogurt)?

- ◯ NEVER

- ◯ 1–6 times per year
- ◯ 7–11 times per year
- ◯ 1 time per month
- ◯ 2–3 times per month
- ◯ 1 time per week
- ◯ 2 times per week
- ◯ 3–4 times per week
- ◯ 5–6 times per week
- ◯ 1 time per day
- ◯ 2 or more times per day

109. How often did you eat **cottage cheese** (including low-fat)?

- ◯ NEVER

- ◯ 1–6 times per year
- ◯ 7–11 times per year
- ◯ 1 time per month
- ◯ 2–3 times per month
- ◯ 1 time per week
- ◯ 2 times per week
- ◯ 3–4 times per week
- ◯ 5–6 times per week
- ◯ 1 time per day
- ◯ 2 or more times per day

110. How often did you eat **cheese** (including low-fat; including on cheeseburgers or in sandwiches or subs)?

- ◯ NEVER **(GO TO QUESTION 111)**

- ◯ 1–6 times per year
- ◯ 7–11 times per year
- ◯ 1 time per month
- ◯ 2–3 times per month
- ◯ 1 time per week
- ◯ 2 times per week
- ◯ 3–4 times per week
- ◯ 5–6 times per week
- ◯ 1 time per day
- ◯ 2 or more times per day

Question 111 appears in the next column.

110a. How often was the cheese you ate **light** or **low-fat cheese**?

- ◯ Almost never or never
- ◯ About 1/4 of the time
- ◯ About 1/2 of the time
- ◯ About 3/4 of the time
- ◯ Almost always or always

111. How often did you eat **frozen yogurt, sorbet,** or **ices** (including low-fat or fat-free)?

- ◯ NEVER

- ◯ 1–6 times per year
- ◯ 7–11 times per year
- ◯ 1 time per month
- ◯ 2–3 times per month
- ◯ 1 time per week
- ◯ 2 times per week
- ◯ 3–4 times per week
- ◯ 5–6 times per week
- ◯ 1 time per day
- ◯ 2 or more times per day

112. How often did you eat **ice cream, ice cream bars,** or **sherbet** (including low-fat or fat-free)?

- ◯ NEVER **(GO TO QUESTION 113)**

- ◯ 1–6 times per year
- ◯ 7–11 times per year
- ◯ 1 time per month
- ◯ 2–3 times per month
- ◯ 1 time per week
- ◯ 2 times per week
- ◯ 3–4 times per week
- ◯ 5–6 times per week
- ◯ 1 time per day
- ◯ 2 or more times per day

112a. How often was the ice cream you ate **light**, **low-fat**, or **fat-free ice cream** or **sherbet**?

- ◯ Almost never or never
- ◯ About 1/4 of the time
- ◯ About 1/2 of the time
- ◯ About 3/4 of the time
- ◯ Almost always or always

113. How often did you eat **pudding or custard**?

- ◯ NEVER

- ◯ 1–6 times per year
- ◯ 7–11 times per year
- ◯ 1 time per month
- ◯ 2–3 times per month
- ◯ 1 time per week
- ◯ 2 times per week
- ◯ 3–4 times per week
- ◯ 5–6 times per week
- ◯ 1 time per day
- ◯ 2 or more times per day

114. How often did you eat **cake** (including low-fat or fat-free)?

- ◯ NEVER

- ◯ 1–6 times per year
- ◯ 7–11 times per year
- ◯ 1 time per month
- ◯ 2–3 times per month
- ◯ 1 time per week
- ◯ 2 times per week
- ◯ 3–4 times per week
- ◯ 5–6 times per week
- ◯ 1 time per day
- ◯ 2 or more times per day

Over the past 12 months...

115. How often did you eat **cookies** or **brownies** (including low-fat or fat-free)?

- ◯ NEVER
- ◯ 1–6 times per year
- ◯ 7–11 times per year
- ◯ 1 time per month
- ◯ 2–3 times per month
- ◯ 1 time per week
- ◯ 2 times per week
- ◯ 3–4 times per week
- ◯ 5–6 times per week
- ◯ 1 time per day
- ◯ 2 or more times per day

116. How often did you eat **doughnuts, sweet rolls, Danish,** or **pop-tarts**?

- ◯ NEVER
- ◯ 1–6 times per year
- ◯ 7–11 times per year
- ◯ 1 time per month
- ◯ 2–3 times per month
- ◯ 1 time per week
- ◯ 2 times per week
- ◯ 3–4 times per week
- ◯ 5–6 times per week
- ◯ 1 time per day
- ◯ 2 or more times per day

117. How often did you eat **sweet muffins** or **dessert breads** (including low-fat or fat-free)?

- ◯ NEVER
- ◯ 1–6 times per year
- ◯ 7–11 times per year
- ◯ 1 time per month
- ◯ 2–3 times per month
- ◯ 1 time per week
- ◯ 2 times per week
- ◯ 3–4 times per week
- ◯ 5–6 times per week
- ◯ 1 time per day
- ◯ 2 or more times per day

118. How often did you eat **fruit crisp, cobbler,** or **strudel**?

- ◯ NEVER
- ◯ 1–6 times per year
- ◯ 7–11 times per year
- ◯ 1 time per month
- ◯ 2–3 times per month
- ◯ 1 time per week
- ◯ 2 times per week
- ◯ 3–4 times per week
- ◯ 5–6 times per week
- ◯ 1 time per day
- ◯ 2 or more times per day

119. How often did you eat **pie**?

- ◯ NEVER **(GO TO QUESTION 120)**
- ◯ 1–6 times per year
- ◯ 7–11 times per year
- ◯ 1 time per month
- ◯ 2–3 times per month
- ◯ 1 time per week
- ◯ 2 times per week
- ◯ 3–4 times per week
- ◯ 5–6 times per week
- ◯ 1 time per day
- ◯ 2 or more times per day

Question 120 appears in the next column.

119a. How often was the pie you ate **fruit pie** (such as apple, cherry, peach, blueberry, or others)?

- ◯ Almost never or never
- ◯ About 1/4 of the time
- ◯ About 1/2 of the time
- ◯ About 3/4 of the time
- ◯ Almost always or always

120. How often did you eat **chocolate candy**?

- ◯ NEVER
- ◯ 1–6 times per year
- ◯ 7–11 times per year
- ◯ 1 time per month
- ◯ 2–3 times per month
- ◯ 1 time per week
- ◯ 2 times per week
- ◯ 3–4 times per week
- ◯ 5–6 times per week
- ◯ 1 time per day
- ◯ 2 or more times per day

121. How often did you eat **other candy**?

- ◯ NEVER
- ◯ 1–6 times per year
- ◯ 7–11 times per year
- ◯ 1 time per month
- ◯ 2–3 times per month
- ◯ 1 time per week
- ◯ 2 times per week
- ◯ 3–4 times per week
- ◯ 5–6 times per week
- ◯ 1 time per day
- ◯ 2 or more times per day

122. How often did you eat **eggs, egg whites,** or **egg substitutes** (NOT counting eggs in baked goods and desserts)? *(Please include eggs in salads, quiche, and souffles.)*

- ◯ NEVER **(GO TO QUESTION 123)**
- ◯ 1–6 times per year
- ◯ 7–11 times per year
- ◯ 1 time per month
- ◯ 2–3 times per month
- ◯ 1 time per week
- ◯ 2 times per week
- ◯ 3–4 times per week
- ◯ 5–6 times per week
- ◯ 1 time per day
- ◯ 2 or more times per day

122a. How often were the eggs you ate **egg substitutes**?

- ◯ Almost never or never
- ◯ About 1/4 of the time
- ◯ About 1/2 of the time
- ◯ About 3/4 of the time
- ◯ Almost always or always

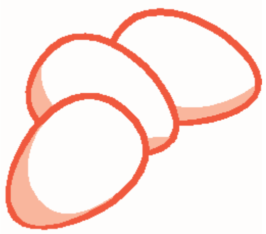

Question 123 appears on the next page.

Over the past 12 months...

122b. How often were the eggs you ate **egg whites only**?

- ◯ Almost never or never
- ◯ About 1/4 of the time
- ◯ About 1/2 of the time
- ◯ About 3/4 of the time
- ◯ Almost always or always

122c. How often were the eggs you ate **regular whole eggs**?

- ◯ Almost never or never
- ◯ About 1/4 of the time
- ◯ About 1/2 of the time
- ◯ About 3/4 of the time
- ◯ Almost always or always

122d. How often were the eggs you ate part of **egg salad**?

- ◯ Almost never or never
- ◯ About 1/4 of the time
- ◯ About 1/2 of the time
- ◯ About 3/4 of the time
- ◯ Almost always or always

123. How many cups of **coffee,** caffeinated or decaffeinated, did you drink?

◯ NONE **(GO TO QUESTION 124)**

- ◯ Less than 1 cup per month
- ◯ 1–3 cups per month
- ◯ 1 cup per week
- ◯ 2–4 cups per week
- ◯ 5–6 cups per week
- ◯ 1 cup per day
- ◯ 2–3 cups per day
- ◯ 4–5 cups per day
- ◯ 6 or more cups per day

123a. How often was the coffee you drank **decaffeinated**?

- ◯ Almost never or never
- ◯ About 1/4 of the time
- ◯ About 1/2 of the time
- ◯ About 3/4 of the time
- ◯ Almost always or always

124. How many glasses of **ICED tea,** caffeinated or decaffeinated, did you drink?

◯ NONE **(GO TO QUESTION 125)**

- ◯ Less than 1 cup per month
- ◯ 1–3 cups per month
- ◯ 1 cup per week
- ◯ 2–4 cups per week
- ◯ 5–6 cups per week
- ◯ 1 cup per day
- ◯ 2–3 cups per day
- ◯ 4–5 cups per day
- ◯ 6 or more cups per day

Question 125 appears in the next column.

124a. How often was the iced tea you drank **decaffeinated** or **herbal tea**?

- ◯ Almost never or never
- ◯ About 1/4 of the time
- ◯ About 1/2 of the time
- ◯ About 3/4 of the time
- ◯ Almost always or always

125. How many cups of **HOT tea**, caffeinated or decaffeinated, did you drink?

◯ NONE **(GO TO QUESTION 126)**

- ◯ Less than 1 cup per month
- ◯ 1–3 cups per month
- ◯ 1 cup per week
- ◯ 2–4 cups per week
- ◯ 5–6 cups per week
- ◯ 1 cup per day
- ◯ 2–3 cups per day
- ◯ 4–5 cups per day
- ◯ 6 or more cups per day

125a. How often was the hot tea you drank **decaffeinated** or **herbal tea**?

- ◯ Almost never or never
- ◯ About 1/4 of the time
- ◯ About 1/2 of the time
- ◯ About 3/4 of the time
- ◯ Almost always or always

126. How often did you add **sugar** or **honey** to your coffee or tea?

◯ NEVER

- ◯ Less than 1 time per month
- ◯ 1–3 times per month
- ◯ 1 time per week
- ◯ 2–4 times per week
- ◯ 5–6 times per week
- ◯ 1 time per day
- ◯ 2–3 times per day
- ◯ 4–5 times per day
- ◯ 6 or more times per day

127. How often did you add **artificial sweetener** to your coffee or tea?

◯ NEVER

- ◯ Less than 1 time per month
- ◯ 1–3 times per month
- ◯ 1 time per week
- ◯ 2–4 times per week
- ◯ 5–6 times per week
- ◯ 1 time per day
- ◯ 2–3 times per day
- ◯ 4–5 times per day
- ◯ 6 or more times per day

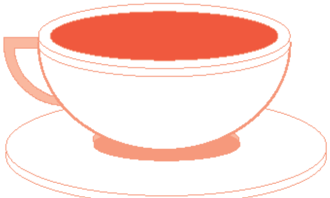

Over the <u>past 12 months</u>...

128. How often was **non-dairy creamer** added to your coffee or tea?

- ◯ NEVER **(GO TO QUESTION 129)**
- ◯ Less than 1 time per month
- ◯ 1–3 times per month
- ◯ 1 time per week
- ◯ 2–4 times per week
- ◯ 5–6 times per week
- ◯ 1 time per day
- ◯ 2–3 times per day
- ◯ 4–5 times per day
- ◯ 6 or more times per day

128a. What kind of **non-dairy creamer** did you usually use?

- ◯ Regular powdered
- ◯ Low-fat or fat-free powdered
- ◯ Regular liquid
- ◯ Low-fat or fat-free liquid

129. How often was **cream** or **half and half** added to your coffee or tea?

- ◯ NEVER
- ◯ Less than 1 time per month
- ◯ 1–3 times per month
- ◯ 1 time per week
- ◯ 2–4 times per week
- ◯ 5–6 times per week
- ◯ 1 time per day
- ◯ 2–3 times per day
- ◯ 4–5 times per day
- ◯ 6 or more times per day

130. How often was **milk** added to your coffee or tea?

- ◯ NEVER **(GO TO QUESTION 131)**
- ◯ Less than 1 time per month
- ◯ 1–3 times per month
- ◯ 1 time per week
- ◯ 2–4 times per week
- ◯ 5–6 times per week
- ◯ 1 time per day
- ◯ 2–3 times per day
- ◯ 4–5 times per day
- ◯ 6 or more times per day

130a. What kind of **milk** was usually added to your coffee or tea?

- ◯ Whole milk
- ◯ 2% milk
- ◯ 1% milk
- ◯ Skim, nonfat, or 1/2% milk
- ◯ Evaporated or condensed (canned) milk
- ◯ Soy milk
- ◯ Rice milk
- ◯ Raw, unpasteurized milk
- ◯ Other

Question 131 appears in the next column.

131. How often was **sugar** or **honey** added to foods you ate? (Please do not include sugar in coffee, tea, other beverages, or baked goods.)

- ◯ NEVER
- ◯ 1–6 times per year
- ◯ 7–11 times per year
- ◯ 1 time per month
- ◯ 2–3 times per month
- ◯ 1 time per week
- ◯ 2 times per week
- ◯ 3–4 times per week
- ◯ 5–6 times per week
- ◯ 1 time per day
- ◯ 2 or more times per day

132. How often did you eat **margarine** on breads, bagels, English muffins, other muffins, pancakes, or waffles?

- ◯ NEVER **(GO TO QUESTION 133)**
- ◯ 1–6 times per year
- ◯ 7–11 times per year
- ◯ 1 time per month
- ◯ 2–3 times per month
- ◯ 1 time per week
- ◯ 2 times per week
- ◯ 3–4 times per week
- ◯ 5–6 times per week
- ◯ 1 time per day
- ◯ 2 or more times per day

132a. How often was the margarine you ate on these breads **low-fat** or **fat-free**?

- ◯ Almost never or never
- ◯ About 1/4 of the time
- ◯ About 1/2 of the time
- ◯ About 3/4 of the time
- ◯ Almost always or always

133. How often did you eat **butter** on breads, bagels, English muffins, other muffins, pancakes, or waffles?

- ◯ NEVER **(GO TO QUESTION 134)**
- ◯ 1–6 times per year
- ◯ 7–11 times per year
- ◯ 1 time per month
- ◯ 2–3 times per month
- ◯ 1 time per week
- ◯ 2 times per week
- ◯ 3–4 times per week
- ◯ 5–6 times per week
- ◯ 1 time per day
- ◯ 2 or more times per day

133a. How often was the butter you ate on these breads **low-fat** or **fat-free**?

- ◯ Almost never or never
- ◯ About 1/4 of the time
- ◯ About 1/2 of the time
- ◯ About 3/4 of the time
- ◯ Almost always or always

Question 134 appears on the next page.

3/8" SPINE PERF

Over the past 12 months...

134. How often did you eat **margarine** on potatoes, cooked vegetables, rice, grains, or beans?

◯ NEVER **(GO TO QUESTION 135)**

◯ 1–6 times per year
◯ 7–11 times per year
◯ 1 time per month
◯ 2–3 times per month
◯ 1 time per week
◯ 2 times per week
◯ 3–4 times per week
◯ 5–6 times per week
◯ 1 time per day
◯ 2 or more times per day

134a. How often was the margarine you ate on these cooked potatoes, cooked vegetables, rice, grains, or beans **low-fat** or **fat-free**?

◯ Almost never or never
◯ About 1/4 of the time
◯ About 1/2 of the time
◯ About 3/4 of the time
◯ Almost always or always

135. How often did you eat **butter** on potatoes, cooked vegetables, rice, grains, or beans?

◯ NEVER **(GO TO QUESTION 136)**

◯ 1–6 times per year
◯ 7–11 times per year
◯ 1 time per month
◯ 2–3 times per month
◯ 1 time per week
◯ 2 times per week
◯ 3–4 times per week
◯ 5–6 times per week
◯ 1 time per day
◯ 2 or more times per day

135a. How often was the butter you ate on these cooked potatoes, cooked vegetables, rice, grains, or beans **low-fat** or **fat-free**?

◯ Almost never or never
◯ About 1/4 of the time
◯ About 1/2 of the time
◯ About 3/4 of the time
◯ Almost always or always

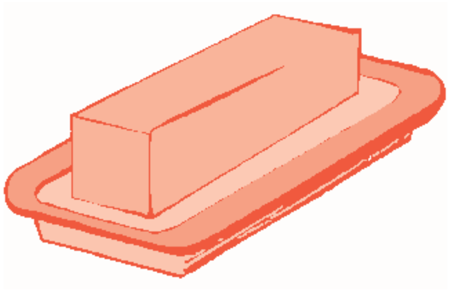

Question 136 appears in the next column.

136. How often did you eat **mayonnaise** as a spread or as part of food mixtures?

◯ NEVER **(GO TO QUESTION 137)**

◯ 1–6 times per year
◯ 7–11 times per year
◯ 1 time per month
◯ 2–3 times per month
◯ 1 time per week
◯ 2 times per week
◯ 3–4 times per week
◯ 5–6 times per week
◯ 1 time per day
◯ 2 or more times per day

136a. How often was the mayonnaise you ate **low-fat** or **fat-free**?

◯ Almost never or never
◯ About 1/4 of the time
◯ About 1/2 of the time
◯ About 3/4 of the time
◯ Almost always or always

137. How often did you eat **cream cheese**?

◯ NEVER **(GO TO QUESTION 138)**

◯ 1–6 times per year
◯ 7–11 times per year
◯ 1 time per month
◯ 2–3 times per month
◯ 1 time per week
◯ 2 times per week
◯ 3–4 times per week
◯ 5–6 times per week
◯ 1 time per day
◯ 2 or more times per day

137a. How often was the cream cheese you ate **low-fat** or **fat-free**?

◯ Almost never or never
◯ About 1/4 of the time
◯ About 1/2 of the time
◯ About 3/4 of the time
◯ Almost always or always

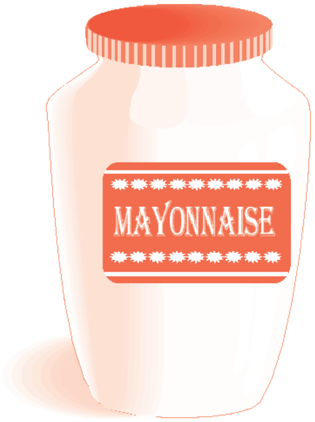

Question 138 appears on the next page.

Over the past 12 months...

138. How often did you eat **sour cream**?

◯ NEVER **(GO TO QUESTION 139)**

◯ 1–6 times per year
◯ 7–11 times per year
◯ 1 time per month
◯ 2–3 times per month
◯ 1 time per week
◯ 2 times per week
◯ 3–4 times per week
◯ 5–6 times per week
◯ 1 time per day
◯ 2 or more times per day

138a. How often was the sour cream you ate **low-fat** or **fat-free**?

◯ Almost never or never
◯ About 1/4 of the time
◯ About 1/2 of the time
◯ About 3/4 of the time
◯ Almost always or always

Question 139 appears in the next column.

139. How often did you eat foods with **oils added** or with **oils used in cooking** (do not include baked goods or salads)?

◯ NEVER

◯ 1–6 times per year
◯ 7–11 times per year
◯ 1 time per month
◯ 2–3 times per month
◯ 1 time per week
◯ 2 times per week
◯ 3–4 times per week
◯ 5–6 times per week
◯ 1 time per day
◯ 2 or more times per day

139a. What kind of **oils** do you **usually eat**? ***(Mark all that apply.)***

◯ Olive
◯ Corn
◯ Canola/rapeseed
◯ Other

Thank you very much for completing this questionnaire! Because we want to be able to use all the information you have provided, we would greatly appreciate it if you would please take a moment to review each page making sure that you:

- ***Did not skip any pages,***
- ***Completely blackened-in each answer, and***
- ***Completely erased any changes you may have made.***

If found, please return to:

Janice Hall
Westat
1650 Research Blvd
Rockville, MD 20850

3/8" SPINE PERF

DE Mark Reflex® forms by NCS Pearson EW-244004-3:654321 HR06 Printed in U.S.A.
